# Dopamine Receptor D3 Expression Is Altered in CD4^+^ T-Cells From Parkinson's Disease Patients and Its Pharmacologic Inhibition Attenuates the Motor Impairment in a Mouse Model

**DOI:** 10.3389/fimmu.2019.00981

**Published:** 2019-05-01

**Authors:** Daniela Elgueta, Francisco Contreras, Carolina Prado, Andro Montoya, Valentina Ugalde, Ornella Chovar, Roque Villagra, Claudio Henríquez, Miguel A. Abellanas, María S. Aymerich, Rarael Franco, Rodrigo Pacheco

**Affiliations:** ^1^Laboratorio de Neuroinmunología, Fundación Ciencia & Vida, Santiago, Chile; ^2^Departamento de Ciencias Biológicas, Facultad de Ciencias de la Vida, Universidad Andres Bello, Santiago, Chile; ^3^Departamento de Ciencias Neurológicas Oriente, Facultad de Medicina, Universidad de Chile, Santiago, Chile; ^4^Departamento de Bioquímica y Genética, Programa de Neurociencias, Centro de Investigación Médica Aplicada (CIMA), Universidad de Navarra, Pamplona, Spain; ^5^Department of Biochemistry and Molecular Biomedicine, University of Barcelona, Barcelona, Spain; ^6^Centro de Investigación en Red, Enfermedades Neurodegenerativas CiberNed, Instituto de Salud Carlos III, Madrid, Spain

**Keywords:** neuroinflammation, neurodegeneration, Parkinson's disease patients, MPTP mouse model, dopamine receptors, CD4+ T-cells

## Abstract

Neuroinflammation constitutes a fundamental process involved in Parkinson's disease (PD). Microglial cells play a central role in the outcome of neuroinflammation and consequent neurodegeneration of dopaminergic neurons in the substantia nigra. Current evidence indicates that CD4^+^ T-cells infiltrate the brain in PD, where they play a critical role determining the functional phenotype of microglia, thus regulating the progression of the disease. We previously demonstrated that mice bearing dopamine receptor D3 (DRD3)-deficient CD4^+^ T-cells are completely refractory to neuroinflammation and consequent neurodegeneration induced by the administration of 1-methyl-4-phenyl-1,2,3,6-tetrahydropyridine (MPTP). In this study we aimed to determine whether DRD3-signalling is altered in peripheral blood CD4^+^ T-cells obtained from PD patients in comparison to healthy controls (HC). Furthermore, we evaluated the therapeutic potential of targeting DRD3 confined to CD4^+^ T-cells by inducing the pharmacologic antagonism or the transcriptional inhibition of DRD3-signalling in a mouse model of PD induced by the chronic administration of MPTP and probenecid (MPTPp). *In vitro* analyses performed in human cells showed that the frequency of peripheral blood Th1 and Th17 cells, two phenotypes favoured by DRD3-signalling, were significantly increased in PD patients. Moreover, naïve CD4^+^ T-cells obtained from PD patients displayed a significant higher Th1-biased differentiation in comparison with those naïve CD4^+^ T-cells obtained from HC. Nevertheless, DRD3 expression was selectively reduced in CD4^+^ T-cells obtained from PD patients. The results obtained from *in vivo* experiments performed in mice show that the transference of CD4^+^ T-cells treated *ex vivo* with the DRD3-selective antagonist PG01037 into MPTPp-mice resulted in a significant reduction of motor impairment, although without significant effect in neurodegeneration. Conversely, the transference of CD4^+^ T-cells transduced *ex vivo* with retroviral particles codifying for an shRNA for DRD3 into MPTPp-mice had no effects neither in motor impairment nor in neurodegeneration. Notably, the systemic antagonism of DRD3 significantly reduced both motor impairment and neurodegeneration in MPTPp mice. Our findings show a selective alteration of DRD3-signalling in CD4^+^ T-cells from PD patients and indicate that the selective DRD3-antagonism in this subset of lymphocytes exerts a therapeutic effect in parkinsonian animals dampening motor impairment.

## Introduction

Several lines of evidence have indicated that neuroinflammation plays a pivotal role in the development of Parkinson's disease (PD) (1). Microglial cells constitute the central players in neuroinflammation, thereby their functional phenotype determines whether surrounding neurons survive or die. In this regard, depending on the integration of molecular cues, microglial cells may acquire either a neurotoxic or a neuroprotective phenotype, which are known as M1 and M2, respectively (2).

Growing evidence in human and animal models has shown the generation of nitrated forms of α-synuclein in the substantia nigra (SN) of individuals with PD (3–5), which is mainly contained in protein inclusions called Lewy bodies. Of note, the nitration of α-synuclein, which is a consequence of the oxidative stress, results in the generation of neo-antigens (1). Furthermore, studies in mice and recently in humans, have shown that oxidised α-synuclein constitutes a major antigen for the T-cell-mediated immune response involved in PD (4, 6–8). In this regard, it has been shown that nitrated α-synuclein generated in the SN is captured and presented by antigen-presenting-cells (APCs) in cervical lymph nodes to naive CD4^+^ T-cells with specificity to this neo-antigen. Once activated, CD4^+^ T-cells acquire inflammatory phenotypes, such as T-helper-1 (Th1) and Th17, then they infiltrate the SN where microglial cells act as local APCs presenting nitrated α-synuclein-derived antigens on class II MHC, thus re-stimulating T-cells (4, 9–11). Re-stimulated CD4^+^ T-cells produce high local levels of IFN-γ and TNF-α, thus promoting further inflammatory activation of microglial cells (M1-microglia) (2, 12, 13). Activated M1-microglia produces several neurotoxic and inflammatory mediators, including reactive oxygen and nitrogen species, which in turn induce neuronal death and further generation of oxidised and nitrated proteins (1). Thus, this mechanism constitutes a vicious cycle, which results in chronic neuroinflammation and represents the engine of the progression of neurodegeneration. Of note, several studies have shown that CD4^+^ T-cells deficiency results in a complete protection of neurodegeneration in mouse models of PD (4, 13, 14), thus indicating that inflammatory CD4^+^ T-cell response is required to promote neurodegeneration of the nigrostriatal pathway.

During last 15 years, several studies have shown dopamine as a major regulator of inflammation (15–17). In this regard, it has been consistently demonstrated that dopaminergic signalling mediated by low-affinity dopamine receptors, including dopamine receptors D1 (DRD1) and DRD2 exerts anti-inflammatory effects in several experimental systems (15, 16, 18). On the other hand, recent studies addressing the role of dopaminergic regulation of CD4^+^ T-cells have shown genetic and pharmacologic evidence indicating that the stimulation of high-affinity dopamine receptors, including DRD3 and DRD5, favours the acquisition of Th1 and Th17 phenotypes, respectively, thus promoting inflammation (19–21).

Since dopaminergic neurons are the main cells affected in PD, dopamine levels are strongly reduced in the brain of PD patients and animal models (14, 22). Thereby, dopaminergic signalling mediated by low-affinity dopamine receptors is favoured in the nigrostriatal pathway of healthy individuals, whilst the selective stimulation of high-affinity dopamine receptors is promoted in PD (17). In this regard, our previous results have shown that DRD3-signalling in CD4^+^ T-cells plays a fundamental role in the development of neurodegeneration in a mouse model of PD induced by the administration of 1-methyl-4-phenyl-1,2,3,6-tetrahydropyridine (MPTP) (13). Accordingly, the genetic deficiency of DRD3 strongly limited the acquisition of the inflammatory potential of CD4^+^ T-cells infiltrating the SN of MPTP-treated mice, abrogating neurodegeneration of the nigrostriatal pathway. Furthermore, the systemic administration of a DRD3-antagonist resulted in a significant attenuation of both neurodegeneration and motor impairment in MPTP-treated mice (23). According to the relevance of DRD3-signalling in animal models of PD, it has been shown a significant association of PD-progression with the reduction of the *Drd3*-transcription in peripheral blood mononuclear cells (PBMCs) obtained from PD patients (24). Thus, current evidence suggests that DRD3-signalling in lymphocytes plays a relevant role favouring the development of PD in animal models and human individuals.

In this study, we addressed the question of whether DRD3 expression is altered in CD4^+^ T-cells obtained from PD patients and how it is associated with the inflammatory phenotypes of these cells. Furthermore, we evaluated the therapeutic potential of the inhibition of DRD3-signalling confined to CD4^+^ T-cells using an animal model of PD induced by the chronic administration of MPTP.

## Materials and Methods

### Human Subjects

Forty-one patients from both genders (22 females and 19 males) who meet Diagnostic Criteria of the Brain Bank of the Society of Parkinson's Disease in the UK (UK PDSBB) were recruited from Hospital del Salvador. Demographic information was collected and summarized in Table 1. Cognitive impairment was evaluated by Montreal Cognitive Assessment (MoCA) test and disease severity was determined with both Unified Parkinson's Disease Rating Scale (UPDRS) and Hoehn and Yahr stages. The functional capacity was obtained as the Schwab and England score. Patients with acute inflammatory or infectious diseases, with chronic inflammatory or autoimmune diseases, with hepatic damage, renal damage, fasting hyperglycemia, or cancer and other diseases that might produce altered immunity were excluded from this study. Thirty-eight age-matched healthy controls (HC) chosen under the same exclusion criteria were included in the study. Venous blood samples were obtained in universal tubes containing heparin. Tubes were subsequently coded and stored at room temperature until processing, which occurred within 2 h after collection.

**Table 1:** Demographic features and disease activity of Parkinson's disease patients.

Gender (Female/Male)	41 (22/19)
Age	65.6 ± 12.2
MoCA test^a^	27 ± 3.2
UPDRS^b^	31 ± 21.2
Hoehn and Yahr^c^	2.1 ± 0.97
Schwab and England^d^	80 ± 18.8

a *Montreal Cognitive Assessment (scale 0–30)*.

b *Unified Parkinson's Disease Rating Scale (scale 0–199)*.

c *Modified Hoehn and Yahr scale (scale 0–5)*.

d *Schwab and England activities of daily living scale (%)*.

### Flow Cytometry Analysis of Peripheral Blood Immune Cells From Human Individuals

Human PBMCs were obtained immediately after the extraction of heparinized blood from HC or PD patients using Ficoll-PaqueTM Plus (Ge Healthcare). PBMCs were immediately analysed or activated with anti-human CD3 monoclonal antibody (mAb; 2 μg/ml, Biolegend) and anti-human CD28 mAb (2 μg/ml, Biolegend) in medium XVIVO-10 (Lonza) containing 1% autologous serum for 3d at 37°C and 5% CO_2_. The expression of DRD3 was analysed in different CD4^+^ T-cell subsets, including total CD4^+^ T-cells (CD3^+^CD4^+^), naive CD4^+^ T-cells (CD3^+^CD4^+^CD45RA^+^CD45RO^−^) and effector/memory (CD3^+^CD4^+^CD45RA^−^CD45RO^+^). DRD3 expression was also analysed in B cells and natural killer (NK). For this purpose immunostaining for surface markers was performed using the following fluorophore-conjugated mAbs: Brilliant violet 421-conjugated anti-CD3 mAb (1:100, Biolegend), FITC-conjugated anti-CD4 mAb (1:100, Biolegend), PECy7-conjugated anti-CD45RO mAb (1:100), Biolegend), APC-Cy7-conjugated anti-CD45RA mAb (1:100, Biolegend), PE-Cy7-conjugated anti-CD56 mAb (1:100, Biolegend), APC-Cy7-conjugated anti-CD19 mAb (1:100, Biolegend) and PE-Cy5-conjugated anti-CD25 mAb (1:100, Biolegend). To determine DRD3 expression we used a primary polyclonal antibody (pAb) anti-DRD3 IgG antibody developed in rabbit (2 μg/ml, Abcam), and a secondary PE-conjugated goat anti-rabbit IgG (Santa Cruz Biotechnology). As an isotype control, irrelevant rabbit polyclonal IgG (2μg/ml, Abcam) was used instead the anti-DRD3 pAb. To study T-cell phenotypes, resting or activated PBMCs were re-stimulated in the presence of 50 ng/ml of phorbol 12-myristate 13-acetate (PMA), 1 μg/ml ionomycin and 5 μg/ml Brefeldin A for 3 h at 37°C and intracellular cytokine or transcription factor staining was analysed in CD4^+^ T-cells. To analyse the extent of T-cell differentiation to the Th1 phenotype, naive CD4^+^ T-cells were purified from PBMCs with the Naive T Cell isolation Kit, Human MACS (Miltenyi Biotec). Afterward, cells were incubated (10^6^ cells/ml) with anti-human CD3 mAb (1μg/ml), anti-human CD28 mAb (2μg/ml), recombinant IL-2 (5μg/ml), recombinant IL-12 (2.5 ng/ml) and anti-human IL-4 mAb (12.5 ng/ml) all from Biolegend, in XVIVO-10 medium containing 1% autologous serum for 5d. Then, cells were re-stimulate with PMA, ionomycin and brefeldin A during 3 h and the frequency of Th1, Th17, and Tregs phenotypes was analysed by intracellular immunostaining of IFN-γ, IL-17, and Foxp3 respectively in the CD4^+^ T-cell population. For intracellular staining the following mAbs were used: PE-Cy7-conjugated anti-IFN-γ mAb (1:100, Biolegend), APC-Cy7-conjugated anti-IL-17 mAb (1:100, Biolegend) and PE-conjugated anti-Foxp3 mAb (1:100, Biolegend).

### Animals

Ten-to-twelve weeks old C57BL/6 mice were used for all *in vivo* experiments. Wild-type (WT) and *Foxp3*^*gfp*^ reporter C57BL/6 mice were purchased from The Jackson Laboratory (Bar Harbor, ME). C57BL/6 *Drd3*^−/−^(DRD3KO) mice were kindly donated by Dr. Marc Caron (25). *Foxp3*^*gfp*^ *Drd3*^−/−^ were generated by crossing parental mouse strains. We confirmed this new strain to be transgenic and Drd3-deficient by flow cytometry analysis of blood cells and PCR of genomic DNA, respectively. Five mice per cage were housed at 21°C in a humidity-controlled environment, on a12/12h light/dark cycle with lights on at 8 a.m., with *ad libitum* access to food and water. All mice were maintained and manipulated according to institutional guidelines at the pathogen-free facility of the Fundación Ciencia & Vida.

### MPTPp Intoxication and Treatments With PG01037

Animals were treated as outlined in Figures 2A, **5A**. Groups received 10 intraperitoneal (i.p.) injections of MPTP hydrochloride (20 mg/kg in saline; Toronto Research Chemicals INC, Toronto, ON, Canada) and probenecid (250 mg/kg in saline; Life Technologies, Oregon, USA), administered twice a week throughout 5 weeks. In all groups receiving MPTP (or the vehicle) and probenecid, both compounds were administered in two consecutive injections during the early morning. Some experimental groups received the i.v. transference of *ex vivo* manipulated CD4^+^ T-cells (as described below) and in other cases mice received the i.p. administration of PG01037 (30 mg/kg; Tocris Bioscience) as indicated in figure legends.

### Viral Transduction

For initial testing of the efficacy of different short hairpin RNA (shRNA) directed to *Drd3* transcription, we generated HEK293T cells overexpressing stably DRD3. For this purpose, HEK293T cells were transfected with lentiviral vectors codifying for the reporter gene red fluorescent protein (RFP) followed by a 2A sequence, puromycin resistance gene and *Drd3*; the whole construct under the control of the CMV promoter (pLenti-GIII-CMV-RFP-2A-Puro-DRD3). Cells were transfected in the presence of turbofect (Thermo Scientific) and 48h later, puromycin (3 μg) was added and cells were grown for 28d. RFP^+^ cells were isolated by cell-sorting and then used to test the efficacy of four different shRNA for *Drd3* transcription (shDrd3 1-4). Afterward, HEK293T overexpressing DRD3 (3.5 × 10^5^ cells per point) were transfected with lentiviral vectors codifying for different versions of shDrd3 or an scrambled shRNA, followed by green fluorescent protein (GFP) reporter gene (piLenti-shRNA-GFP). Forty-eight hours later, cells were lysed and the levels of *Drd3* transcripts were quantified by qRT-PCR.

For silencing DRD3 expression in CD4^+^ T-cells, we used the retroviral vector pBullet (26), which was kindly provided by Dr. Hinrich Abken. We inserted a region encoding GFP, U6 promoter, shRNA against DRD3 (shDrd3-3; 5′-TGC CCT CTC CTC TTT GGT TTC AAC ACA AC-3′) and H1 promoter, into pBullet vector via NcoI and SalI restriction sites (Genscript, *Pisca*taway, NJ). pBullet vector drives the expression of the entire construct by the CMV promoter upstream the NcoI site. This vector was transfected into Phoenix-AMPHO cells and GFP^+^ cells were purified by cell sorting to generate a stable cell line producing shDRD3 retrovirus (RV-shDRD3) in the supernatant as described before (20). Total CD4^+^ T-cells were activated with α-CD3ε mAb 1 μg/ ml, α-CD28 mAb 1 μg/ ml, IL-2 10 ng/ ml in RPMI medium containing 5% FBS in 6-well plates at 37°C and 5% CO_2_ for 96 h. Cells were infected with retroviral particles at 24 and 48 h of incubation. Infection was carried out by spinoculating cells with retrovirus in retronectin-coated plates (Takara Bio, Japan). As a non-silencing control, we transduced CD4^+^ T-cells with a control vector (RV-Control) codifying just for GFP. At day 5 of culture, cells were restimulated and transduction efficiency was determined (GFP^+^ cells) in CD4^+^ T-cells by flow cytometry.

### Quantitative RT-PCR

Levels of *Drd3* transcripts were quantified as described previously (20). Briefly, total RNA extracted from cells using the Total RNA EZNA kit (Omega Bio-Tek), was DNase-digested using the TURBO DNA-free kit (Ambion) and 1 μg of RNA was used to synthesize cDNA utilizing M-MLV reverse transcriptase (Life Technologies). Quantitative gene expression analysis was performed using Brilliant II SYBR Green QPCR Master Mix (Agilent). Primers were used at a concentration of 0.5 μM. Expression of *Drd3* was normalised to *Gapdh*. The sequences of the primers used are the following: *Drd3*, forward 5n′-GAA CTC CTT AAG CCC CAC CAT-3′ and reverse 5′-GAA GGC CCC GAG CAC AAT-3′; and *Gapdh*, forward 5′-TCC GTG TTC CTA CCC CCA ATG-3′ and reverse 5′-GAG TGG GAG TTG CTG TTG AAG-3′.

### Transference of *ex vivo* Manipulated CD4^+^ T-Cells

Anti-CD3 mAb (100 ng/well) in PBS was pre-incubated in 96-well plates during 16h at 4°C and then washed twice with PBS. Total splenic CD4^+^ T-cell were isolated by using a negative selection kit (Miltenyi) and incubated (3 × 10^6^ cells per well) in anti-CD3-coated 96-well plates containing soluble anti-CD28 mAbs (100 ng/well). Immediately after inducing T-cell activation, in some cases cells were treated with 20 nM PG01037 for 24 h and then i.v. injected (4 × 10^5^, 7 × 10^5^, or 1 × 10^6^ cells/mouse) into recipient mice. In other cases, CD4^+^ T-cells were transduced with RV-shDRD3 or RV-Control and then GFP^+^ cells were purified by cell-sorting and subsequently i.v. transferred (4 × 10^5^ cells/mouse) into recipient mice.

### Coat-Hanger Test

To determine the motor performance, we used the coat-hanger test, which has been validated for detection of motor dysfunctions (27, 28). Briefly, we used a steel coat hanger (diameter: 2 mm, length: 40 cm) suspended at a height of 35 cm from a cushioned surface. The surface of the coat hanger was marked with regular sections of 5 cm each. The mice were placed in the middle of the hanger and the time taken to move from the middle of the hanger to an extreme was recorded (extreme latency). In addition, the number of sections by which mice moved though after the first 60 seconds was also determined (# sections).

### Beam Test

As a second test to evaluate motor performance we used a simplified version of the beam test previously described (29). Briefly, we used a horizontal beam 25 cm length and 3 cm width. The beam surface was covered by a metallic grid (1 cm^2^). Mice were videotaped while traversing the grid-surface beam from one of the extreme of the beam to the opposite extreme, where the home-cage was located. Two days of training were performed for habituation to the task. The number of errors (# errors) was quantified by watching the videos in slow-motion mode. An error was defined as when a forelimb or hindlimb slipped through the grid and became visible between the grid and the beam surface or on the side of the grid during a forward movement.

### Tissue Processing

Animals were sacrificed by transcardial perfusion 48 h after the last MPTPp injection. For histological techniques, mice were anesthetised with an overdose of 5% isoflurane (Sigma-Aldrich) and transcardially perfused with 4% paraformaldehyde (Merck, Darmstadt, Germany) in 0.125 M phosphate buffered saline (PBS, pH 7.4). Brains were removed and cryoprotected for 48 h in 20% glycerin and 2% DMSO in PBS. For flow cytometry analysis, mice were transcardially perfused with PBS instead paraformaldehyde, brains were rapidly removed, dissected, and immediately processed for flow cytometry as indicated below.

### Histological Techniques and Quantification

Immunohistochemistry was performed on free-floating sections (40 μm thick) and for a given experiment all sections were processed at the same time with the respective primary antibody. Sections were washed with PBS and endogenous peroxidase activity was inactivated by 30 min incubation with 0.03% H_2_O_2_ in methanol (Sigma-Aldrich). After washing three times with PBS, the tissue was incubated for 40 min with blocking solution [4% goat serum, 0.05% Triton X-100 (Sigma-Aldrich) and 4% BSA (Merck, Darmstadt, Germany) in PBS], and exposed overnight to the primary antibodies diluted in blocking solution at room temperature. The primary antibodies used were: rabbit anti-tyrosine hydroxylase pAb (TH, 1:1000; Millipore, Temecula, CA, USA), rat anti-dopamine transporter pAb (anti-DAT, 1:500; Millipore), rabbit anti-GFAP antibody (1:500; abcam [EPR1034Y], Cambridge, UK) and rabbit anti-Iba1 antibody (1:1000; abcam [EPR16588], Cambridge, UK). For colorimetric immunohistochemistry, antibody binding was detected by incubating sections with biotinylated goat anti-rabbit pAb (1:500; Jackson ImmunoResearch Laboratories, West Gore, PA, USA) or biotinylated goat anti–rat pAb (1:500; Jackson ImmunoResearch Laboratories, West Gore, PA, USA) in blocking solution for 2 h at room temperature. The biotinylated antibodies were detected with peroxidase-conjugated avidin (1:5000; Sigma-Aldrich) for 90 min at room temperature followed by incubation with 0.05% diaminobenzidine (Sigma-Aldrich) in 0.03% H_2_O_2_/Trizma-HCl buffer (pH 7.6). Sections were mounted on glass slides in a 0.2% solution of gelatin in 0.05 M Tris (pH 7.6) (Sigma-Aldrich). The mean number of TH^+^ neurons from six SN *pars compacta* (SNpc) sections (separated by 120 μm between each other) per mouse was quantified under light microscopy at a magnification of 200X, and the total area of SNpc was calculated using ImageJ software (National Institutes of Health, Bethesda, MD). Density of dopaminergic neurons was expressed as the number of TH^+^ neurons per area (mm^2^) in the SNpc. The intensity of immunostaining of dopaminergic terminals in the striatum was evaluated within the TH- or DAT–immunoreactive area (optical density) and was quantified using ImageJ software. To evaluate the extent of astrogliosis, the mean of GFAP-associated immunoreactivity was analysed in areas of interest (660 μm x 877 μm) in five striatum sections per mouse and quantified as the integrated density using the Image-J software. To determine the extent of activated microglia, the mean number of Iba-1^high^ reactive microglia displaying ameboid shape was quantified in areas of interest of 660 μm x 877 μm in five striatum sections per mouse.

### Flow Cytometry Analysis of Mouse T-Cells

Deep cervical lymph nodes and brain sections that contain mid-brain and striatum from MPTPp-treated mice were minced and then disaggregated using Collagenase Type IV 1mg/ml (Gibco, New York, USA) and DNase I 0.25 mg/mL (Roche, Mammheim, Germany). After enzymatic disaggregation, cells were passage through 70 μm-pore cell-strainer to obtain a single-cell suspension. The cell suspension was centrifuged in a gradient of Percoll TM GE Healthcare (Fermelo Biotec) 70%/40%. The mononuclear cells were extracted from the interface and resuspended in RPMI 1640 medium supplemented with 10% FBS. Then, cells were re-stimulated with 50 ng/ml PMA; 1 μg/ml ionomycin and 5 μg/ml Brefeldin A for 3 h at 37°C. To evaluate the phenotype of T-cells, the mononuclear cells were stained with fluorochromes-coupled mAbs directed to surface markers, fixed with formaldehyde 1% and permeabilized with Staining Buffer Factor Set eBioscience^TM^ Foxp3/Transcriptm (Thermo Fisher Scientific). Afterwards, cells were stained with fluorochromes-coupled mAbs directed to cytokines and transcriptional factors. The expression of cytokines and transcription factors in different T-cell subsets was analysed by flow cytometry (FACSCantoII, BD Bioscience). For surface or intracellular immunostaining, anti-CD4, anti-TCRγδ, anti-IFN-γ, anti-IL-17, anti-Foxp3, and anti-RORγt fluorochrome-conjugated mAbs were used at a dilution of 1:300 (all from Biolegend). For *in vitro* T-cell activation assays, effector CD4^+^ T-cells (Teff; GFP^−^) and regulatory CD4^+^ T-cells (Treg; GFP^+^) were isolated from the spleen of *Foxp3*^*gfp*^ reporter mice by cell sorting using a FACS Aria II (BD), obtaining purities over 98%. All *in vitro* experiments were performed using complete RPMI medium (supplemented with 10% FBS, 2 mM L-Glutamine, 100 U/mL Penicillin, 100 μg/mL Streptomycin and 50 μM β-mercaptoethanol). To assess activation, cells were stimulated for 6d with 50 ng/well of plate-bound anti-CD3 mAb and 2 μg/mL soluble anti-CD28 mAb on flat-bottom 96-well plates (Thermo Scientific). IL-2 (10 ng/mL) was added to the culture at days 0, 3, and 5. To force the Th1 differentiation naive CD25^−^CD4^+^ T-cells were activated in the presence of 20 ng/mL IL-12, 10 ng/mL IL-2, and 5 μg/mL anti-IL-4 for 4d. All analyses were performed in living cells using the Zombie Aqua (Zaq) fixable viability kit (Biolegend) in the ZAq^−^ population. Flow cytometry analysis was performed using a FACS Canto II (BD). Data were analysed using the FlowJo software (Tree Star).

### Statistical Analysis

All values were expressed as mean ± SEM. Differences in means between PD patients and HC groups were analysed by 2-tailed unpaired Student's *t*-test. Comparisons between different experimental groups in MPTPp experiments were performed by one-way ANOVA followed by the multi-comparison Tukey's *post-hoc* test. Correlations between different parameters were analysed by Pearson's test when data was normally distributed or by Spearman's test when data was not normally distributed. Normal distribution was determined by Shapiro–Wilk test. *P*-value ≤ 0.05 was considered significant. Analyses were performed with GraphPad Prism 6 software.

### Study Approval

The study performed with human individuals conforms to the principles outlined in the Declaration of Helsinki, the study protocol was approved by the local Ethics Committee of the Hospital del Salvador, Santiago (Chile), and all the participants signed a written informed consent before enrollment. All procedures performed in animals were approved by and complied with regulations of the Institutional Animal Care and Use Committee at Fundación Ciencia & Vida.

## Results

### DRD3 Expression Is Reduced in CD4^+^ T-Cells Obtained From PD Patients

**Figure 1:**
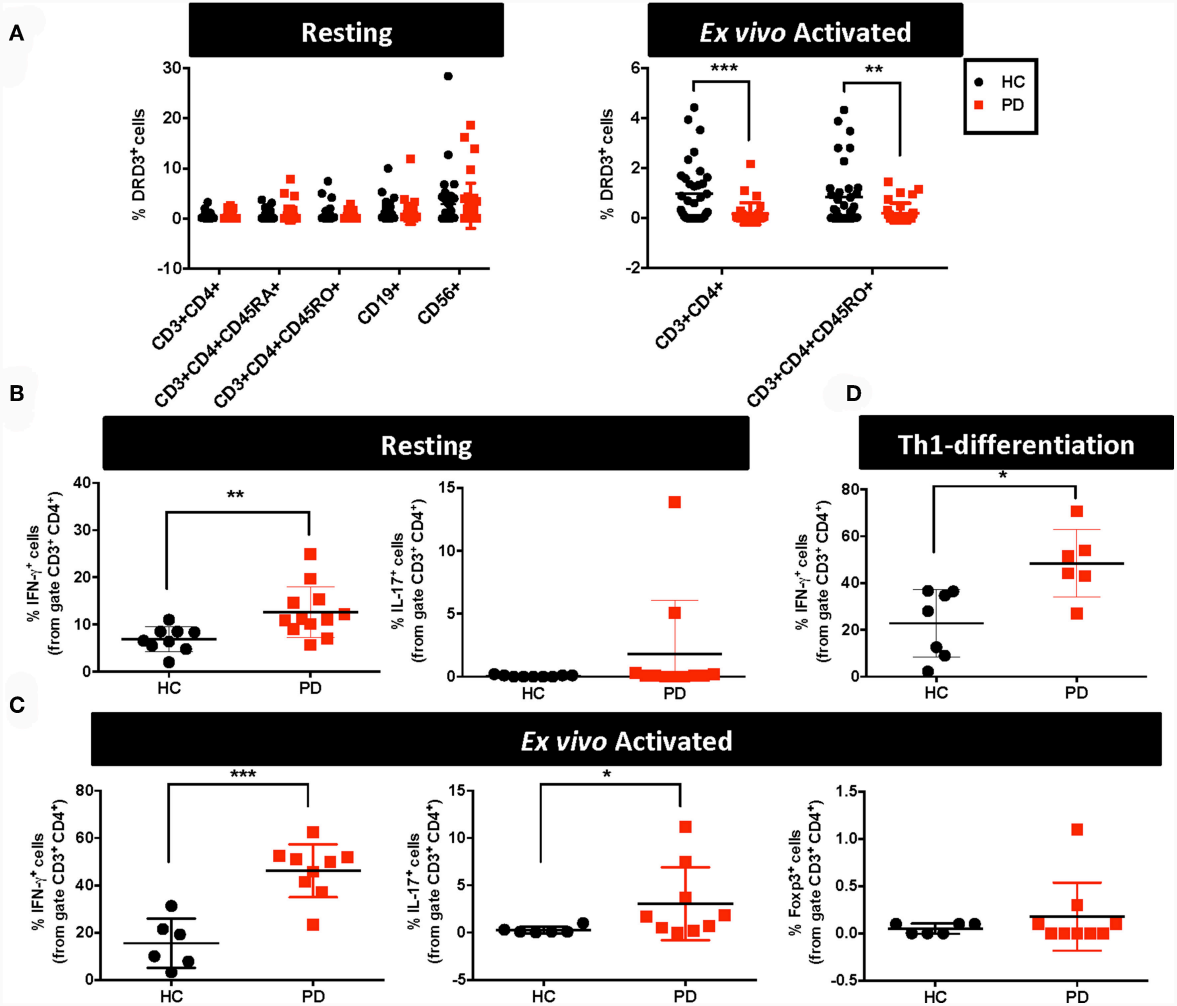
Alterations in the functional phenotype and DRD3 expression in CD4^+^ T-cells obtained from Parkinson's disease patients. **(A–C)** PBMCs were isolated from healthy donors (HC) and Parkinson's Disease patients (PD) and they were immediately analysed or activated with anti-CD3 and anti-CD28 antibodies for 72 h. **(A)** Cells were immunostained for several surface markers and DRD3 expression was analysed in different lymphocyte populations by flow cytometry. Left panel shows the frequency of DRD3^+^ cells in total CD4^+^ T-cells (CD3^+^ CD4^+^), naïve CD4^+^ T-cells (CD3^+^ CD4^+^ CD45RA^+^), memory CD4^+^ T-cells (CD3^+^ CD4^+^ CD45RO^+^), B-cells (CD19^+^), and NK cells (CD56^+^) in resting conditions. Right panel shows the frequency of total CD4^+^ T-cells (CD3^+^ CD4^+^) and memory/effector CD4^+^ T-cells (CD3^+^ CD4^+^ CD45RO^+^) after T-cell activation. Two-tailed unpaired Student *t*-test was used for comparisons between HC (*n* = 38) and PD (*n* = 41). ^**^p < 0.01; ^***^, *p* < 0.0001. **(B)** Resting T-cells or **(C)** T-cells activated with anti-CD3 and anti-CD28 antibodies for 72 h were stimulated with PMA and ionomycin in the presence of brefeldin A for 4 h and IFN-γ (left panels), IL-17 (middle panels), and Foxp3 (right panels) expression was determined by intracellular immunostaining in the CD3^+^ CD4^+^ gated population. Foxp3^+^ cells were undetectable in resting conditions. Two-tailed unpaired Student *t*-test was used for comparisons between HC [*n* = 9 in **(B)**; *n* = 6 in **(C)**] and PD [*n* = 11–12 in **(B)**; *n* = 9 in **(C)]**. ^*^p < 0.05; ^**^p < 0.01; ^***^p < 0.0001. **(D)** Naïve CD4^+^ T-cells (CD3^+^ CD4^+^ CD45RA^+^) T-cells were isolated from PBMCs obtained from HC and PD individuals and then they were incubated in Th1-biased conditions for 5 days. Afterwards, cells were stimulated with PMA and ionomycin in the presence of brefeldin A for 3 h and IFN-γ expression was determined by intracellular immunostaining in the CD3^+^ CD4^+^ gated population. Two-tailed unpaired Student *t*-test was used for comparisons between HC (*n* = 7) and PD (*n* = 6). ^*^p < 0.05.

As stated above, emerging evidence has shown that DRD3-signalling in CD4^+^ T-cells plays a pivotal role favouring the development of PD in animal models (13, 23). Since a previous study found a significant reduction in the levels of *drd3*-transcripts in PBMCs obtained from PD (24), we addressed here the question of whether DRD3 expression, at the level of protein, was altered in CD4^+^ T-cells obtained from PD patients. For this purpose, we analysed DRD3 expression in different lymphocyte populations from PD patient samples displaying different stages of PD progression. Accordingly, we analysed blood samples from 38 HC and 41 PD patients. In these samples we determined DRD3 expression in different CD4^+^ T-cells subsets, including naive CD4^+^ T-cells, memory CD4^+^ T-cells, effector CD4^+^ T-cells and total CD4^+^ T-cells. In addition, we also included the expression of DRD3 in B cells and in natural killer (NK) cells, as we found high levels of DRD3 expression in these lymphocyte populations obtained from healthy donors samples (Figure 1A, left panel). We analysed DRD3 expression in the different lymphocyte subsets by flow cytometry using the gating strategy indicated (Supplementary Figure 1). Interestingly, when DRD3 expression was compared in HC and PD patients, we did not find differences in DRD3 expression in total CD4^+^ T-cells (CD3^+^ CD4^+^), naive CD4^+^ T-cells (CD3^+^ CD45RA^+^ CD4^+^), memory/effector CD4^+^ T-cells (CD3^+^ CD45RO^+^ CD4^+^), B-cells (CD19^+^) and NK cells (CD56^+^) upon resting conditions (Figure 1A, left panel). Conversely, when DRD3 expression was evaluated after *ex vivo* T-cell activation, DRD3 expression was significantly reduced in total CD4^+^ T-cells (CD3^+^ CD4^+^) and in memory/effector CD4^+^ T-cells (CD3^+^ CD4^+^ CD45RO^+^) obtained from PD patients (Figure 1A, right panel). This data indicates a significant down-regulation of DRD3 expression in *ex vivo* activated CD4^+^ T-cells obtained from PD patients. We next attempted to evaluate whether DRD3 down-regulation was associated with PD activity. For this purpose, we analysed the potential correlations between DRD3 expression and the clinical score of PD patients evaluated by different tests, including the UPDRS, the Modified Hoehn and Yahr scale, the Schwab and England score and the MoCA test. Interestingly, we found a significant correlation between DRD3 down-regulation in naïve CD4^+^ T-cells and the UPDRS clinical score (Supplementary Figure 2), although we did not find significant associations of disease activity with the DRD3 down-regulation in activated total or memory/effector CD4^+^ T-cells (Supplementary Figure 3).

### CD4^+^ T-Cells Obtained From PD Patients Display an Increased Percentage of Pro-inflammatory Phenotypes and Biased Th1-Differentiation

DRD3-signalling in CD4^+^ T-cells has been consistently associated to Th1 and Th17 mediated immunity (13, 19, 20). Furthermore, Th1 and Th17 have been proven to be the inflammatory phenotypes of CD4^+^ T-cells driving neuroinflammation and consequent neurodegeneration of dopaminergic neurons in animal models of PD (6, 13). On the other hand, it has been demonstrated that suppressive activity of Tregs is able to dampen T-cell mediated inflammation in PD models, thus attenuating neurodegeneration (6, 30, 31). For these reasons, we next aimed to determine potential alterations in the percentage of inflammatory T-cell phenotypes Th1 and Th17 as well as in the extent of the anti-inflammatory T-cell phenotype, Treg, obtained from PD patients in comparison to HC. Accordingly, in a subgroup of PD and HC we first analysed the functional phenotypes in resting T-cells. For this purpose, immediately after isolation from fresh blood samples, PBMC were stimulated with PMA and ionomycin for 4h and cytokine production and the expression of key transcription factors were analysed by intracellular immunostaining followed by flow cytometry analysis. These analyses in “resting” conditions (after just a short period of stimulation) were performed to have an idea of the frequency of inflammatory and anti-inflammatory phenotypes contained in the population of memory and effector T-cells. The results show that PD individuals presented 2-fold higher Th1 frequency in resting CD4^+^ T-cells in comparison with HC (Figure 1B). On the other hand, Th17 frequency in resting CD4^+^ T-cells was similar in PD and HC, whilst Tregs were not detectable in these conditions (Figure 1B and data not shown). To allow the expansion of T-cells, we next analysed the frequency of relevant functional phenotypes of CD4^+^ T-cells after the activation with anti-CD3 and anti-CD28 antibodies for 3d. Then, activated T-cells were re-stimulated with PMA and ionomycin during the last 4h and cytokine production and the expression of transcription factors were quantified by intracellular immunostaining followed by flow cytometry analysis. The results show about 3-fold increase in the frequency of both Th1 and Th17 phenotypes in *ex vivo* activated CD4^+^ T-cells obtained from PD patients in comparison with those obtained from HC (Figure 1C). Of note, in these conditions Tregs were detectable, although no differences were observed between PD patients and HC (Figure 1C). We also analysed the potential association of Th1, Th17, Treg, or total CD4^+^ T-cells frequencies in peripheral blood of PD patients with the severity of the disease, however we did not find any significant correlation (Supplementary Figure 4). Since we observed a higher difference in Th1 frequency between PD and HC after *ex vivo* T-cell activation (Figure 1C) than when compared in resting conditions (Figure 1B), we wondered whether naive CD4^+^ T-cells differentiating to Th1 phenotype could be contributing to this higher Th1 frequency. To address this possibility, we performed experiments in which naive CD4^+^ T-cells were first isolated by cell-sorting, cultured in Th1-skewed conditions and then the extent of Th1 differentiation was compared between PD patients and HC. Interestingly, these results show a 2-fold increase of Th1 differentiation in PD patients in comparison with HC (Figure 1D). Taken together these results indicate that CD4^+^ T-cells in PD patients present higher frequencies of pro-inflammatory phenotypes and naive cells display a skewed Th1-differentiation.

### The Transference of CD4^+^ T-Cells Treated *ex vivo* With a Selective DRD3-Antagonist Exerts a Therapeutic Effect Attenuating Motor Impairment in MPTPp-Treated Mice

**Figure 2:**
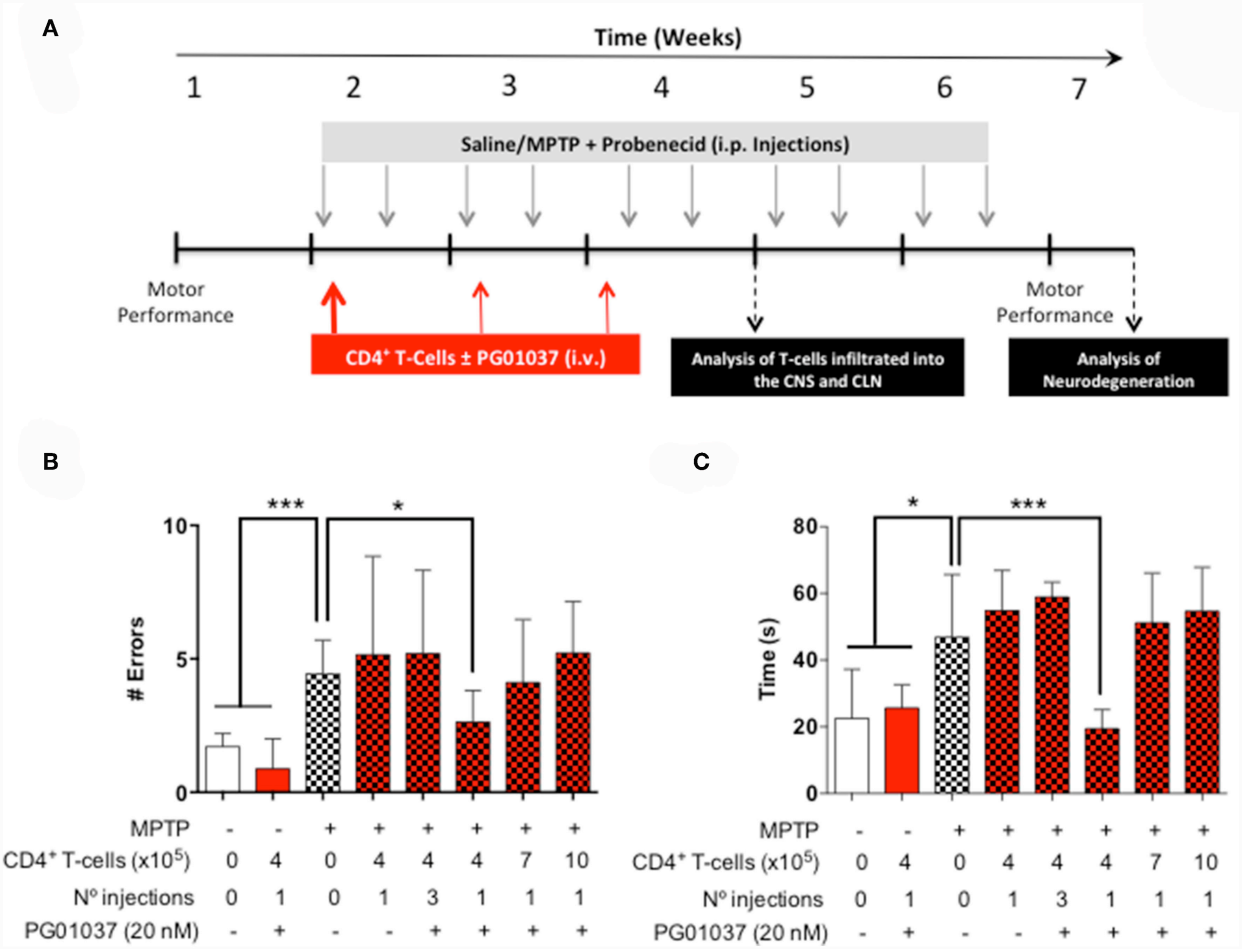
Evaluation of the therapeutic potential of CD4^+^ T-cells treated *ex vivo* with a DRD3 antagonist in the motor performance of MPTPp-treated mice. **(A)** Experimental design: Control animals (without MPTPp treatment) were treated with saline, probenecid, and with or without the i.v. injection of CD4^+^ T-cells treated *ex vivo* with PG01037. MPTPp animals received 10 i.p. injections with MPTP (20 mg/kg) and probenecid (250 mg/kg) during weeks 2–6 (grey arrows). CD4^+^ T-cells (4 × 10^5^, 7 × 10^5^, or 10 × 10^5^ per mouse) were treated with or without PG01037 (20 nM) and then i.v. injected in experimental animals 1 day after the first MPTPp injection (bold red arrow). In some cases, animals received 3 injections of CD4^+^ T-cells separated by 1 week intervals (bold and thin red arrows). T-cell infiltration was analysed after 3 weeks of MPTPp-treatment. Neurodegeneration was analysed 1 week after the last MPTPp injection. Motor performance was analysed the week before beginning with MPTPp administration to distribute experimental groups with homogeneous motor performance and then it was evaluated again 16 h after the last MPTPp injection in the Beam-test **(B)** and in the coat-hanger test **(C)**. Experimental groups receiving i.v. injections of CD4^+^ T-cells are indicated in red bars. Data represents the mean with the SEM. One-way ANOVA followed by Tukey's multiple comparison *post hoc* test were used to determine statistical differences: ^*^p < 0.05 ^***^p < 0.001, *n* = 5–17 mice per group.

Since a prominent role of DRD3-signalling in CD4^+^ T-cells has been observed in the development of PD in mouse models (13, 32), and the systemic DRD3-antagonsim has been proven to attenuate neurodegeneration and motor impairment in different animal models of PD (23), we next aimed to test the therapeutic potential of the selective inhibition of DRD3-confined to CD4^+^ T-cells. For this purpose, we used an animal model of PD induced by the chronic administration of MPTP and probenecid (MPTPp), which results in both, loss of dopaminergic neurons of the nigrostriatal pathway and a significant motor impairment (33). To exert a selective DRD3-antagonism in CD4^+^ T-cells, these cells were pre-incubated *ex vivo* with PG01037 (hereinafter called CD4^+^/PG01037) (34) and then transferred into MPTPp-treated mice. Of note, DRD3-antagonism mediated by PG01037 20 nM attenuated the potentiation of Th1-differentiation exerted by the selective stimulation of DRD3 with dopamine 50 nM (Supplementary Figure 5). Accordingly, we performed a titration of the number of injections and the number of CD4^+^ T-cells per injection able to exert a significant therapeutic effect on MPTPp-treated mice. For this purpose, CD4^+^ T-cells were incubated with PG01037 20 nM *ex vivo* and then, 4x10^5^, 7x10^5^ or 10x10^5^ CD4^+^ T-cells per mouse were transferred in a single i.v. injection into MPTPp mice. Moreover, another group of mice was treated with three injections (separated by 7d between) of 4x10^5^ CD4^+^ T-cells per mouse each (Figure 2A). We also used a control group of mice that received CD4^+^ T-cells without *ex vivo* treatment with PG01037. In all experimental groups we determined the therapeutic potential at the level of motor impairment and neurodegeneration and the extent of participation of different T-cell subsets into the midbrain and cervical lymph nodes (CLN). Notably, the results show that only a single injection of 4 × 10^5^ CD4^+^/PG01037, but not single injections of 7 × 10^5^ or 10 × 10^5^ or three injections of 4 × 10^5^ CD4^+^/PG01037 per mouse, exerted significant attenuation of motor impairment as determined by the beam test (# errors; Figure 2B) and the coat-hanger test (extreme latency; Figure 2C). Thus, these results indicate that the treatment of MPTPp-intoxicated mice with a single injection of 4 × 10^5^ CD4^+^/PG01037 exerts a therapeutic effect at the level of motor impairment in this animal model.

**Figure 3:**
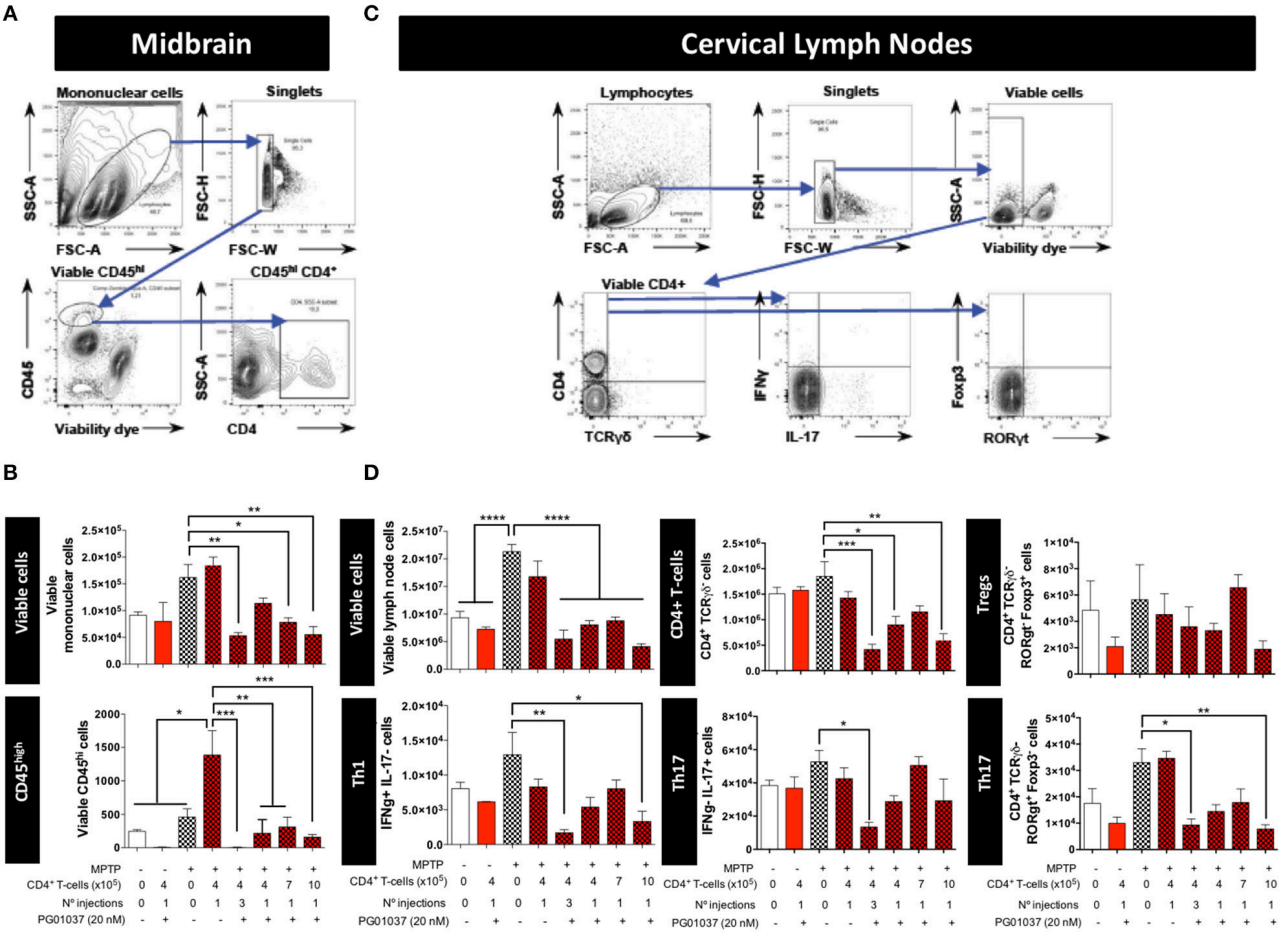
Analysis of T-cell infiltration into the brain and cervical lymph nodes in MPTPp-intoxicated mice after transference of CD4^+^ T-cells treated with PG01037 *ex vivo*. Animals were treated as described in Figure 2A and sacrificed after 3 weeks of MPTPp intoxication (at the end of week number 4 in the scheme of Figure 2A). The frequencies of different inflammatory and anti-inflammatory lymphocyte subsets infiltrating the midbrain **(A,B)** and cervical lymph nodes **(C,D)** were analysed by flow cytometry. **(A,C)** Representative dot plots showing the gating strategy. **(B,D)** absolute numbers per animal of different lymphocyte subsets obtained from the different experimental groups were quantified. Data represent the mean with the SEM. One-way ANOVA followed by Tukey's multiple comparison *post-hoc* test was used to determine statistical differences: ^*^p < 0.05; ^**^p < 0.01; ^***^p < 0.001; ^****^p < 0.0001; *n* = 3 mice per group.

To determine how the different regime of treatment of MPTPp mice with the transfer of *ex vivo* manipulated CD4^+^ T-cells affected the participation of different T-cell subsets into the midbrain and CLN, we first determine the time-point in which neuroinflammation was already evident in this animal model. Accordingly, we evaluated the dynamics of M1 and M2 phenotypes in microglial cells in the brain and the extent of T-cell infiltration in meningeal vessels at different time-points in MPTPp animals. We observed that both, meningeal CD4^+^ T-cells and M1-microglia were already increased after 3 weeks of MPTPp treatment (Supplementary Figure 6) and thereby we chose this time-point to analyse the phenotypes of CD4^+^ T-cells infiltrating the midbrain and CLN. Interestingly, the results show that the single injection of 4 × 10^5^ CD4^+^/PG01037 was the only treatment that did not reduce the number of mononuclear cells infiltrating the midbrain of MPTPp mice (Figures 3A,B), coinciding with the therapeutic effect observed at the level of motor impairment. Conversely, at the level of T-cells infiltrating CLN, all the therapeutic regime of transfer of CD4^+^/PG01037 in MPTPp-intoxicated mice resulted in significant reduction of alive lymph nodes cells (Figures 3C,D). Moreover, single injections of 4 × 10^5^ or 10 × 10^5^ or three injections of 4 × 10^5^ CD4^+^/PG01037, but not the single injection of 7 × 10^5^ CD4^+^/PG01037 significantly attenuated the number of CD4^+^ T-cells infiltrating the CLN in MPTPp mice (Figures 3C,D). Interestingly, only a single injection of 10 × 10^5^ or three injections of 4 × 10^5^ CD4^+^/PG01037 resulted in attenuated number of Th1 and Th17 in the CLN of MPTPp-intoxicated mice (Figures 3C,D). In addition, any of the treatments proven show a significant difference in the extent of Tregs infiltrating CLN in MPTPp mice (Figures 3C,D). Thus, unexpectedly, the only difference at the level of T-cells infiltrating midbrain and CLN that was associated with the selective therapeutic effect at the level of motor impairment was the absence of reduction in the number of total mononuclear cells infiltrating the midbrain of MPTPp mice. To address a potential effect of DRD3-signalling in the viability of CD4^+^ T-cells, we isolated CD4^+^ T-cells from the spleen of WT or DRD3-defficient mice and then were activated for 6d and subsequently the viability was analysed in Teff and Treg cells by flow cytometry. The results show no significant differences in the frequency of living Treg or Teff between both genotypes (Supplementary Figure 7), thus ruling out the possibility that DRD3-signalling affected CD4^+^ T-cells viability.

**Figure 4:**
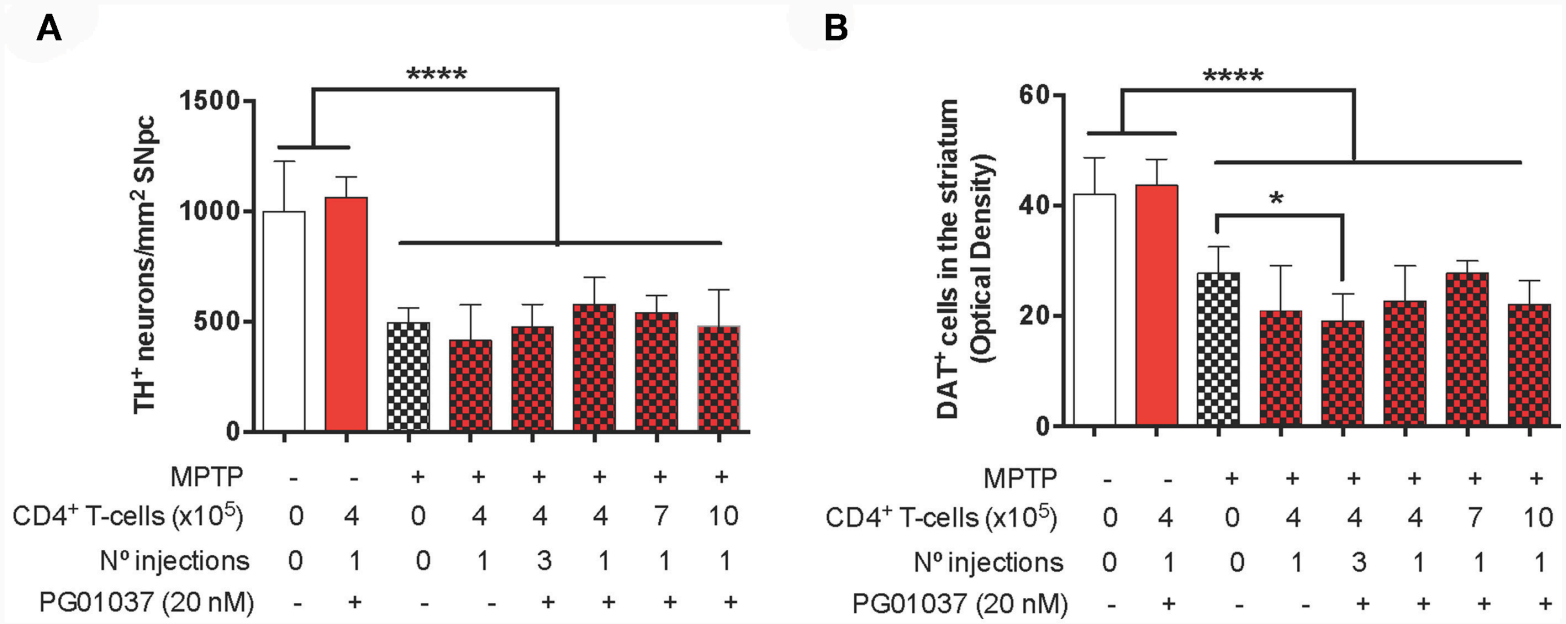
Evaluation of the therapeutic potential at the level of neurodegeneration of DRD3 inhibition in CD4+ T-cells by treatment with a selective antagonist *ex vivo* in MPTPp-treated mice. CD4^+^ T-cells (4 × 10^5^, 7 × 10^5^, or 10 × 10^5^ per mouse) were treated with or without PG01037 (20 nM) and then i.v. injected in experimental animals 1 day after the first MPTPp injection (see Figure 2A). In some cases, animals received 3 injections of CD4^+^ T-cells separated by 1 week intervals. Neurodegeneration was analysed 1 week after the last MPTPp injection. Dopaminergic neurons were quantified by immunohistochemical analysis of tyrosine hydroxylase (TH) in the substantia nigra pars compacta (SNpc) **(A)** and dopamine transporter (DAT) in the striatum **(B)**. Data represent the mean with the SEM. One-way ANOVA followed by Tukey's multiple comparison *post-hoc* test was used to determine statistical differences: ^*^p < 0.05; ^****^p < 0.0001; *n* = 5–8 mice per group.

Finally, to determine the therapeutic potential of the different regime of T-cell transfer at the level of neurodegeneration, we next quantified the loss of dopaminergic neurons in the SN and the extent of dopaminergic terminals in the striatum of experimental mice. For this purpose, after the determination of motor performance, mice were sacrificed and dopaminergic neurons were quantified by immunohistochemical analysis of tyrosine hydroxylase (TH) in the SN *pars compacta* (SNpc) and the density of dopaminergic terminals was evaluated in the striatum by immunohistochemical analysis of dopamine transporter (DAT). Quite unexpected, the results show that the extent of neurodegeneration was not significantly attenuated by any of the regime of *ex vivo* manipulated T-cell transfer (Figure 4). Taken together these results indicate that a single injection of 4 × 10^5^ CD4^+^/PG01037 exerts a therapeutic effect in MPTPp mice attenuating motor impairment but without effect in the extent of neurodegeneration.

### The *ex vivo* Transcriptional Inhibition of *Drd3* in CD4^+^ T-Cells Does Not Exert Any Therapeutic Effect in MPTPp-Treated Mice

**Figure 5:**
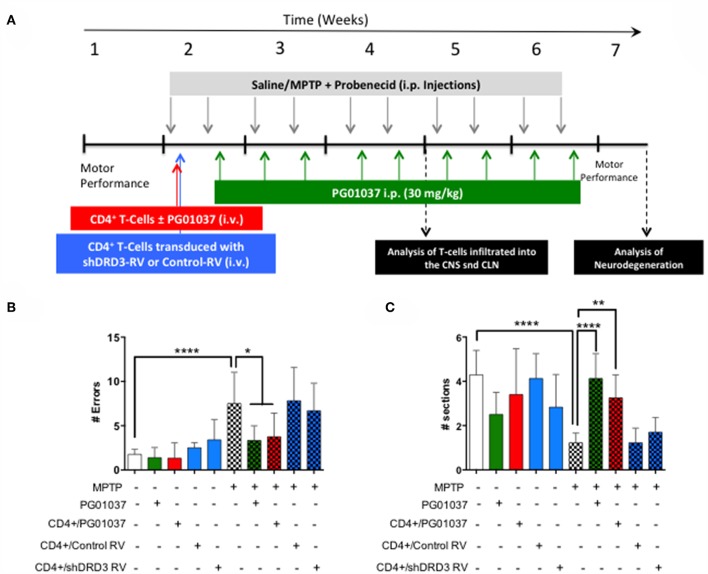
Comparison of the therapeutic potential of the systemic DRD3-antagonistm or the intravenous transference of CD4^+^ T-cells transduced with shRNA for DRD3 or treated with a DRD3 antagonist on the motor performance of MPTPp-treated mice. **(A)** Experimental design: Control animals (without MPTPp treatment) were treated with saline and probenecid alone, or with i.p. PG01037, or the i.v. transference of CD4^+^ T-cells treated with PG01037, transduced with RV-Control or transduced with RV-shDRD3. MPTPp animals received 10 i.p. injections with MPTP (20 mg/kg) and probenecid (250 mg/kg) during weeks 2–6 (grey arrows). CD4^+^ T-cells (4 × 10^5^ cells per mouse) were treated with 20 nM PG01037 (red) or transduced with retroviral particles (MOI 1:1) codifying for RV-Control or RV-shDRD3 (blue) and then i.v. injected in experimental animals 1 day after the first MPTPp injection. Mice treated with systemic DRD3-antagonism (green) received 9 i.p. injections of PG01037 (30 mg/kg) administered 1 day after MPTPp injections starting after the second MPTPp administration. T-cell infiltration was analysed after 3 weeks of MPTPp-treatment. Neurodegeneration was analysed 1 week after the last MPTPp injection. Motor performance was analysed the week before beginning with MPTPp administration to distribute experimental groups with homogeneous motor performance and then it was evaluated again 16 h after the last MPTPp injection in the Beam-test **(B)** and in the coat-hanger test **(C)**. Data represents the mean with the SEM. One-way ANOVA followed by Tukey's multiple comparison *post hoc* test were used to determine statistical differences: ^*^p < 0.05, ^**^p < 0.01, ^****^p < 0.0001, *n* = 5–12 mice per group.

**Figure 6:**
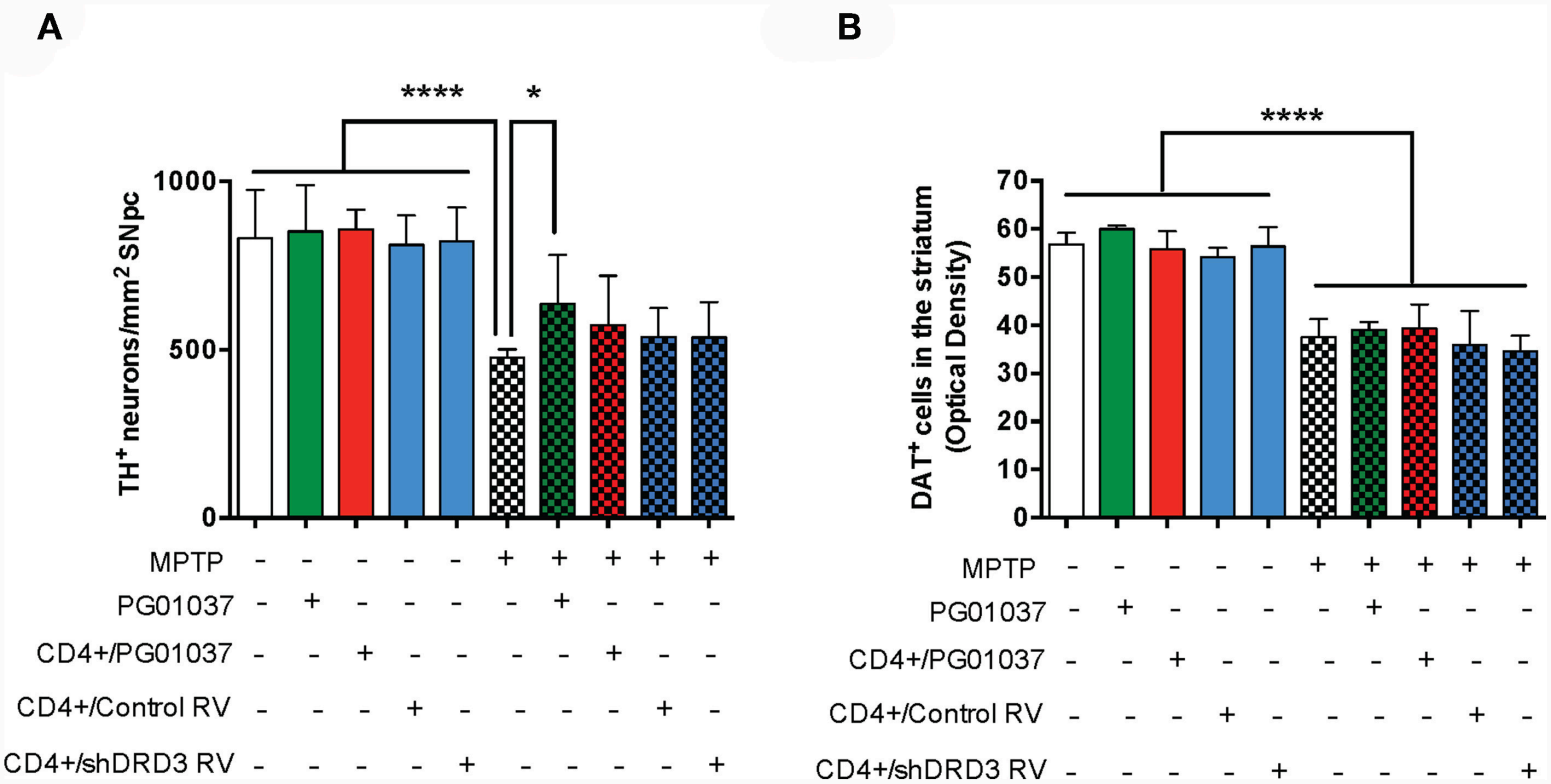
Comparison of the therapeutic potential of the systemic DRD3-antagonistm or the intravenous transference of CD4^+^ T-cells transduced with shRNA for DRD3 or treated with a DRD3 antagonist on neurodegeneration of MPTPp-treated mice. CD4^+^ T-cells (4 × 10^5^ cells per mouse) were treated with 20 nM PG01037 (red) or transduced with retroviral particles (MOI = 1) codifying for RV-Control or RV-shDRD3 (blue) and then i.v. injected in experimental animals 1 day after the first MPTPp injection. Mice treated with systemic DRD3-antagonism (green) received 9 i.p. injections of PG01037 (30 mg/kg) administered 1 day after MPTPp injections starting after the second MPTPp administration (see scheme in Figure 5A). Neurodegeneration was analysed 1 week after the last MPTPp injection. Dopaminergic neurons were quantified by immunohistochemical analysis of tyrosine hydroxylase (TH) in the substantia nigra pars compacta (SNpc) **(A)** and dopamine transporter (DAT) in the striatum **(B)**. Data represent the mean with the SEM. One-way ANOVA followed by Tukey's multiple comparison *post-hoc* test were used to determine statistical differences: ^*^p < 0.05; ^****^p < 0.0001 *n* = 5–7 **(A)** or *n* = 5–11 **(B)** mice per group.

Because the pharmacologic DRD3-antagonism of CD4^+^ T-cells *ex vivo* exerted a therapeutic effect only at the level of motor impairment but not at the level of neurodegeneration we attempted to improve the therapeutic power inducing a more sustained inhibition of DRD3-signalling in CD4^+^ T-cells. For this purpose, we generated retroviral particles codifying for an shRNA to interfere with *Drd3*-transcription (shDRD3). After setting up the transduction protocols, we confirmed that retroviral transduction with the shDRD3 (RV-shDRD3) actually reduced the levels of *Drd3*-transcripts and resulted in impaired production of IFN-γ by CD4^+^ T-cells (Supplementary Figure 8), as described before (20). Afterwards, we performed a set of experiments aimed to compare the therapeutic potency of the transfer of CD4^+^/PG01037, the transfer of CD4^+^ T-cells *ex vivo* transduced with RV-shDRD3 (hereinafter called CD4^+^/RV-shDRD3) or the systemic DRD3-antagonism. For this purpose, MPTPp-intoxicated mice received a single injection of 4 × 10^5^ CD4^+^/PG01037, a single injection of 4 × 10^5^ CD4^+^/RV-shDRD3 or the i.p. administration of PG01037 at 30 mg/kg and the extent of neurodegeneration, motor impairment and T-cell phenotypes in midbrain and CLN were determined (Figure 5A). The results show that only the transfer of CD4^+^/PG01037 or the systemic administration of PG01037, but not the transfer of CD4^+^/RV-shDRD3 exerted a significant attenuation of motor impairment in MPTPp-intoxicated mice reducing the number of errors in the beam test (Figure 5B) and increasing the number of sections travelled in the coat-hanger test (Figure 5C). It is noteworthy that in a previous study using the same animal model of PD, we show that a single i.p. injection of 30 mg/kg PG01037 had no effect in motor impairment (23). Since the remaining PG01037 concentration present in CD4^+^/PG01037 (after the *ex vivo* treatment with 20 nM PG01037 followed by cells washing) is much lower than that present in a single i.p. injection of 30 mg/kg PG01037, it is tempting to rule out that the therapeutic effect exerted by CD4^+^/PG01037 in motor impairment is independent of CD4^+^ T-cells and just due to PG01037. The analysis of T-cell phenotypes in midbrain and CLN did not give any significant differences, despite they show interesting trends for the treatment with systemic PG01037 in decreasing Th1 and Th17 frequencies and increasing Tregs frequency in CLN of MPTPp-intoxicated mice (Supplementary Figure 9). Consistently with previous results (23), the quantification of dopaminergic neurons of the nigrostriatal pathway shows a significant reduction in neuronal loss only when MPTPp-intoxicated mice were treated with systemic PG01037, but not when received the transfer of CD4^+^/PG01037 or CD4^+^/RV-shDRD3 (Figure 6). Together, these results indicate that whereas the systemic DRD3-antagonism exerts a therapeutic effect attenuating neurodegeneration and motor impairment, the transfer of CD4^+^/PG01037 exerts a therapeutic effect only confined to the motor impairment but without effect in neuronal loss. Quite unexpectedly, the transfer of CD4^+^/RV-shDRD3 did not exert any detectable therapeutic effect. A summary of the different therapeutic effects observed for the different treatments in different set of experiments carried out in this study is shown in Table S1.

### The Systemic DRD3-Antagonism as Well as the Transference of CD4^+^ T-Cells Treated *ex vivo* With a Selective DRD3-Antagonist Reduce the Extent of Microglial Activation in MPTPp-Treated Mice

**Figure 7:**
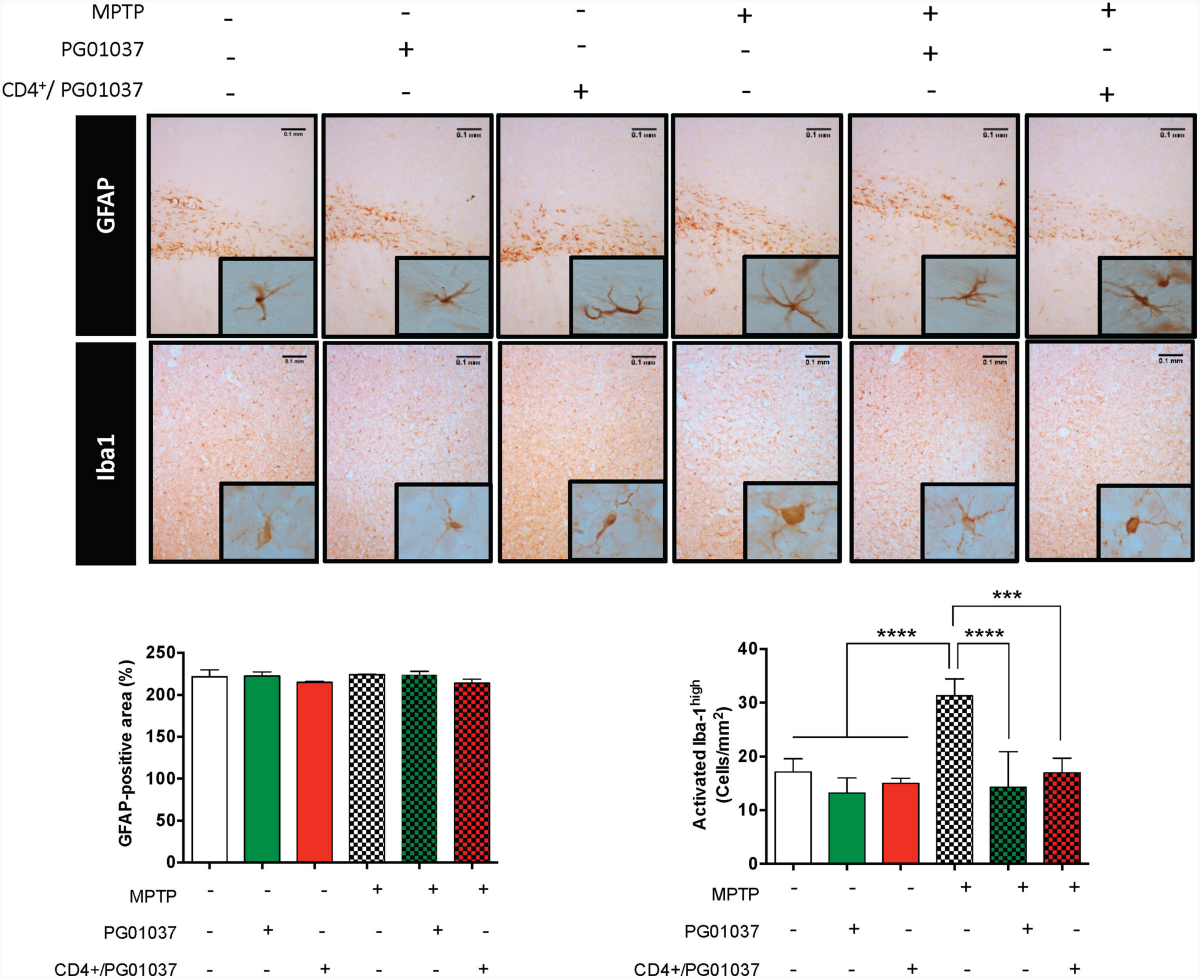
Analysis of neuroinflammation in MPTPp-intoxicated mice upon treatment with systemic DRD3-antagonism or with the intravenous transference of CD4^+^ T-cells treated with a DRD3 antagonist. CD4^+^ T-cells (4 × 10^5^ cells per mouse) were treated with 20 nM PG01037 (red) and then i.v. injected in experimental animals 1 day after the first MPTPp injection. Mice treated with systemic DRD3-antagonism (green) received 9 i.p. injections of PG01037 (30 mg/kg) administered 1 day after MPTPp injections starting after the second MPTPp administration (see scheme in Figure 5A). Neuroinflammation was analysed 1 week after the last MPTPp injection. Astrogliosis was quantified by immunohistochemical analysis of GFAP and microglial activation was quantified by immunohistochemical analysis of Iba1 in the striatum. Representative overview images of GFAP (top panel) and Iba1 (middle panel) immunostaining are shown. Quantification of GFAP-associated density (bottom-left panel) and the number of Iba1^high^ cells per area (bottom-right panel) are shown in the bottom panels. Data represent the mean with the SEM from 3 to 5 mice per group. One-way ANOVA followed by Tukey's multiple comparison *post-hoc* test were used to determine statistical differences: ^***^p < 0.001; ^****^p < 0.0001.

To gain a deeper insight in the mechanism involved in the therapeutic effect exerted by the systemic DRD3-antagonism and by the transfer of CD4^+^/PG01037 in MPTPp-treated mice, we next attempted to analyse how was affected astrocyte and microglial activation. For this purpose, MPTPp-intoxicated mice received a single injection of 4 × 10^5^ CD4^+^/PG01037, or the i.p. administration of PG01037 at 30 mg/kg (as indicated in Figure 5A) and the extent of astrogliosis and microglial activation were evaluated in the striatum by immunohistochemical analyses of GFAP and Iba1, respectively. Unexpectedly, the results show no differences in the degree of astrogliosis among the different experimental groups (Figure 7). On the other hand, both the systemic DRD3-antagonism and the transfer of CD4^+^/PG01037 induced a marked reduction in the extent of microgliosis in MPTPp-treated mice (Figure 7). Thus, these results show that the therapeutic effect observed for systemic DRD3-antagonism at the level of neurodegeneration and motor impairment and for the transfer of CD4^+^/PG01037 at the level of motor impairment involve an attenuation in microglial activation.

## Discussion

Our data here demonstrates a significant and selective reduction of DRD3-expression confined to CD4^+^ T-cells obtained from PD patients. Moreover, our results indicate that the pharmacologic DRD3-antagonism, but not the interference of *Drd3-*trascription in CD4^+^ T-cells *ex vivo* resulted in a therapeutic effect at the level of motor impairment. However, only systemic DRD3-antagonism, but not the transfer of CD4^+^/PG01037, reduced the extent of neurodegeneration of the dopaminergic neurons of the nigrostriatal pathway.

Interestingly, our results show a significant reduction of DRD3 expressed in CD4^+^ T-cells, which could be due to a compensatory mechanism attempting to decrease the inflammatory effect induced by DRD3-stimulation in this lymphocyte population (13). Moreover, this alteration on DRD3 expression in CD4^+^ T-cells obtained from PD patients could represent a useful marker for diagnostic analysis. Of note, Nagai et al., described before that the levels of *Drd3* mRNA were decreased in total PBMCs obtained from PD patients and the degree of this down-regulation was correlated with the stage of disease progression (24). Accordingly, we found a significant correlation between DRD3 down-regulation (at the protein level) in naive CD4^+^ T-cells and the degree of disease activity (see the UPDRS score in Supplementary Figure 2). Of note, we did not find significant associations of disease activity with the DRD3 down-regulation in any other lymphocyte subset analysed, including B-cells, NK cells, resting or activated total CD4^+^ T-cells and activated memory/effector CD4^+^ T-cells (Supplementary Figure 2, 3). Thus, these results suggest a selective association of clinical PD progression with the extent of down-regulation of DRD3 expression selectively on naive CD4^+^ T-cells.

Despite our previous results obtained in mouse models indicate that DRD3-signalling in CD4^+^ T-cells favours the development of PD (13), our results obtained from PD patients show a significant and selective reduction of DRD3 expression in CD4^+^ T-cells obtained from peripheral blood in comparison to those obtained from HC (Figure 1). Thus, this reduction of DRD3 expression in CD4^+^ T-cells obtained from PD patients could be interpreted as an adaptive down-regulation of this pro-inflammatory receptor after a long-term period of chronic inflammation in these patients. Nevertheless, another plausible explanation for this fact can be that simply, CD4^+^ T-cells specific for relevant antigens associated to PD [i.e., nitrated α-synuclein; (1, 35)] would acquire inflammatory phenotypes with high DRD3-expression (20) and they would be just infiltrating the site of inflammation (into the SNpc) but not recirculating in the periphery. This fact would explain why CD4^+^ T-cells expressing low levels of DRD3 would be selectively found in the periphery. This latter hypothesis highlights the advantages that should have an antigen-specific therapy based in CD4^+^ T-cells as a treatment for PD. In this regard, it is expected that a therapy involving the inhibition of DRD3 confined only to those CD4^+^ T-cells specific for relevant antigens associated to PD (i.e., nitrated α-synuclein) would exert a stronger therapeutic potential than those therapeutic approaches tested here involving the inhibition of DRD3-signalling in CD4^+^ T-cells irrespective of their antigen-specificity. Furthermore, an antigen-specific CD4^+^ T-cell based therapy for PD would avoid the multiple potential side-effects exerted by systemic administration of dopaminergic drugs, such as PG01037.

Importantly, we found that naive CD4^+^ T-cells obtained from Chilean PD patients display an increased differentiation toward Th1, a functional phenotype that has been involved in the inflammatory response associated to neurodegeneration in animal models (6, 13). In the same direction, in an study performed in a cohort of 82 Italian PD patients, Kustrimovic *et al*., have performed an analysis of the different functional T-cell phenotypes and have described an Th1-biased immune signature in both, drug-naive or drug-treated PD (36). In addition, another previous study carried out with 40 Italian PD patients has shown a positive correlation between the degree of PD progression with the levels of IFN-γ produced by PBMCs (37). Thereby, together these results suggest that this Th1-skewed differentiation of naive CD4^+^ T-cells is a general feature of PD patients, irrespective of their ethnicity and independent on the administration of dopaminergic drugs.

The present findings indicate that both, the systemic DRD3-antagonism as well as the transfer of CD4^+^/PG01037 attenuated the motor impairment induced by MPTPp-intoxication, however the systemic treatment with PG01037 was the only therapeutic approach that reduced the loss of dopaminergic neurons in the SNpc. This differential therapeutic effect exerted by the systemic DRD3-antagonism in comparison to the transfer of CD4^+^/PG01037 could be due to that systemic administration of PG01037 would be able to block DRD3-signalling not only in CD4^+^ T-cells, but also in astrocytes. In this regard, we have obtained evidence suggesting that DRD3-inhibition in astrocytes favours an anti-inflammatory astrogliosis, which was associated with reduced number of inflammatory microglia (23). Accordingly, in the present study we observed that both the systemic DRD3-antagonsim and the transfer of CD4^+^/PG01037 decreased the number of inflammatory microglia, but without apparent effect in astrocyte activation (Figure 7). In addition to astrocytes and CD4^+^ T-cells, DRD3 has been described to be expressed in other subsets of immune cells that could play a relevant role in neuroinflammation associated to PD, including B-cells, NK cells, and neutrophils (38). In this regard, auto-antibodies recognising Lewy bodies and dopaminergic neurons have been detected in the serum and infiltrated in the brain parenchyma of PD patients (39). Furthermore, the stereotaxic delivery of IgG purified from PD patients induces a significant loss of dopaminergic neurons of the SN in rats in comparison with the effect observed for IgG purified from HC, thus suggesting a relevant role of B-cells in the physiopathology of PD (40). On the other hand, it has been described a significant reduction in the expression of the NK inhibitory receptor NKG2A in PD (41). Moreover, a recent meta-analysis performed with 943 patients indicated an association of PD with increased number of NK cells (42), suggesting a role for NK cells in the physiopathology of this disorder. In addition, Th17 cells, which have been consistently involved in PD (6, 43, 44) exert their effector function mainly by recruiting neutrophils to the site of inflammation, where they release cytotoxic granules inducing directly the death of target cells (45). Thus, DRD3 expressed in neutrophils, B-cells or NK cells could potentially also play a role promoting PD development and progression, nevertheless, further studies are necessaries to address experimentally the relevance and relative contribution of these potential mechanisms in PD.

Intriguingly, the i.v. transfer of CD4^+^/PG01037 into MPTPp-treated mice, reduced the motor impairment but without significant effects in neurodegeneration. This discrepancy could be due to that, by affecting IL-4 and IFN-γ production (20), DRD3-signalling in CD4^+^ T-cells might be involved in the cross-talk between T-cell function and neuronal tasks, irrespective of the neurodegenerative process. In this regard, it has been previously shown that IL-4-produced by CD4^+^ T-cells might regulate the acquisition of spatial memory in the hippocampus (46). Whether DRD3-signalling in CD4^+^ T-cells may affect neural circuitry involved in motor performance or not should be addressed in further studies.

Intriguingly, when we tested the therapeutic potential of the transfer of increasing number of CD4^+^/PG01037 into MPTPp-mice, we did not observe a dose-response curve. Unexpectedly, we obtained a therapeutic effect only with the lower dose of CD4^+^/PG01037 (4 × 10^5^ cells per mouse), but not with higher doses of T-cells (7 × 10^5^ or 10 × 10^5^ cells per mouse). According to these results, it has been previously shown that the transfer of low dose of CD4^+^ T-cells (5 × 10^4^ cells per mouse) exerts a stronger effect than higher doses of CD4^+^ T-cells in an anti-tumour therapy (47). In this regard, it is though that the transfer of lower doses of therapeutic CD4^+^ T-cells allows a stronger expansion *in vivo* after antigen-recognition.

Unexpectedly, whereas the DRD3-antagonsim confined to CD4^+^ T-cells exerted a significant attenuation of motor impairment in MPTPp-treated mice, the transcriptional inhibition of *Drd3* in CD4^+^ T-cells had no effect in motor impairment. This apparent controversy among our results could be due to the different extent of DRD3 inhibition. In this regard, DRD3-antagonism in CD4^+^ T-cells was performed with 20 nM PG01037, a concentration that represents approximately 28-fold its K_i_ (34). Thereby, the conditions used to promote DRD3-antagonism ensure the inhibition of nearly all DRD3 expressed in CD4^+^ T-cells. On the other hand, despite CD4^+^/RV-shDRD3 displayed a significant impairment in the production of IFN-γ (Supplementary Figure 8E), the reduction exerted in the levels of *drd3*-transcripts was about 50% (Supplementary Figure 8D). Thereby, it is likely that this milder effect in DRD3-inhibition exerted by CD4^+^ T-cells transduction with RV-shDRD3 would explain why we could not observe any detectable therapeutic effect at the level of motor impairment or neurodegeneration. At this point, it is important to note that upon T-cell activation, DRD3 expression was sharply reduced in CD4^+^ T-cells (Supplementary Figure 10), although the levels of *drd3* transcripts were increased (20). These results suggest that DRD3-mediated effects are triggered in resting conditions or early after CD4^+^ T-cell activation, but with consequences later in T-cell response. Moreover, since T-cell activation is required to promote retroviral transduction in CD4^+^ T-cells, we could not observe a significant effect of shDRD3 transduction on DRD3 expression in activated CD4^+^ T-cells (Supplementary Figure 10). Thus, a plausible explanation for the lack of therapeutic effect observed for the transfer of CD4^+^ T-cells transduced *ex vivo* with RV-shDRD3 is that an early inhibition of DRD3-signalling in resting CD4^+^ T-cells is necessary to evoke the beneficial effects induced in motor impairment in MPTPp-treated mice.

It is intriguing that our previous study evaluating the therapeutic potential of systemic administration of PG01037 shows a significant increase in anti-inflammatory astrogliosis and attenuation of neurodegeneration of the nigrostriatal pathway at the level of neuronal bodies in the SNpc and at the level of dopaminergic terminals in the striatum (23), however the present study shows just a therapeutic effect at the level of SNpc, but not in the striatum and without effect in astrocyte activation (Figures 4, 7). This discrepancy could be explained by the fact that these two set of experiments were performed in two different animal facilities, and therefore the microbiota composition of experimental mice should be different as well. In this regard, it has been recently demonstrated that gut microbiota has a strong impact in the susceptibility of individuals to develop neurodegeneration and the motor impairment associated to PD in humans and animal models (48).

Finally, it is important to note that the treatments that exerted therapeutic effects here, the systemic PG01037 administration and the i.v. transfer of CD4^+^/PG01037, were administered early during the induction of the disease (24 h after the second MPTPp injection and 24 h after the first MPTPp injection, respectively). Despite these treatments were given before the motor onset, early diagnosis of PD in humans is currently quickly evolving. Indeed, there are some key early symptoms, including REM sleep disorder, olfactory loss (49), and gut-associated issues (50) that together might predict PD manifestation and the development of motor impairment with many years in advance. In this regard, drug-design is currently pointing to stop the progression of neurodegeneration in PD at early stages of diagnosis, earlier than the onset of motor impairment.

## Ethics Statement

The study performed with human individuals conforms to the principles outlined in the Declaration of Helsinki, the study protocol was approved by the local Ethics Committee of the Hospital del Salvador, Santiago (Chile), and all the participants signed a written informed consent before enrollment. All procedures performed in animals were approved by and complied with regulations of the Institutional Animal Care and Use Committee at Fundación Ciencia & Vida.

## Author Contributions

RP designed the study. DE, FC, CP, AM, VU, and OC conducted experiments. DE, FC, CP, AM, VU, OC, CH, and RV acquired data. DE, FC, CP, AM, OC, MAA, MSA, RF, and RP analysed data. MAA and MSA provided new reagents. DE and RP wrote the manuscript.

### Conflict of Interest Statement

The authors declare that the research was conducted in the absence of any financial or non-financial competing interests as defined by this journal, with the exception of a pending patent application describing therapeutic use of selective DRD3-antagonist in Parkinson's disease, and which could be construed as a potential conflict of interest. Authors of said patent present in this paper are: RP, DE, FC, and VU.

## Abbreviations

Ab: Antibody
APC: antigen-presenting cells
DRD*n*: Dopamine receptor *n*
Foxp3: forkhead box P3
GFP: green fluorescent protein
GFAP: Glial Fibrillary Acidic Protein
IFN-γ: interferon γ
IL-*n*: interleukin *n*
mAb: monoclonal Ab
MFI: mean fluorescence intensity
MoCA: Montreal Cognitive Assessment
MPTP: 1-methyl-4-phenyl-1, 2, 3, 6-tetrahydropyridine
pAb: polyclonal Ab
PBMCs: peripheral blood mononuclear cells
PE: phycoerythrin
PD: Parkinson's disease
PMA: phorbol 12-myristate 13-acetate
RAG1: recombination-activating-gen-1
RAG1KO: RAG1 knockout
SN: substantia nigra
SNpc: SN *pars compacta*
Th*n*: T helper *n*
TLRs: Toll like receptors
TLR*n*: Toll like receptor *n*
TNF-α: Tumor Necrosis Factor α
Tregs: regulatory T cells
UPDRS: Unified Parkinson's Disease Rating Scale.
