# Supplementary material for: Dopamine Receptor D3 Expression Is Altered in CD4+ T-Cells From Parkinson's Disease Patients and Its Pharmacologic Inhibition Attenuates the Motor Impairment in a Mouse Model
Source: Front Immunol. 2019 May 1;10:981. doi: 10.3389/fimmu.2019.00981 (PMC6504698; doi:10.3389/fimmu.2019.00981)
Supplement: Supplementary file 1 [file Data_Sheet_1.PDF]

Supplementary Material

Dopamine receptor D3 expression is altered in $CD4^+$ T-cells from Parkinson's disease patients and its pharmacologic inhibition attenuates the motor impairment in a mouse model

Daniela Elgueta, Francisco Contreras, Carolina Prado, Andro Montoya, Valentina Ugalde, Ornella Chovar, Roque Villagra, Claudio Henríquez, Miguel A. Abellanas, María S. Aymerich, Rarael Franco, Rodrigo Pacheco*.

*** Correspondence:** Rodrigo Pacheco; rpacheco@cienciavida.org; rodrigo.pacheco@unab.cl

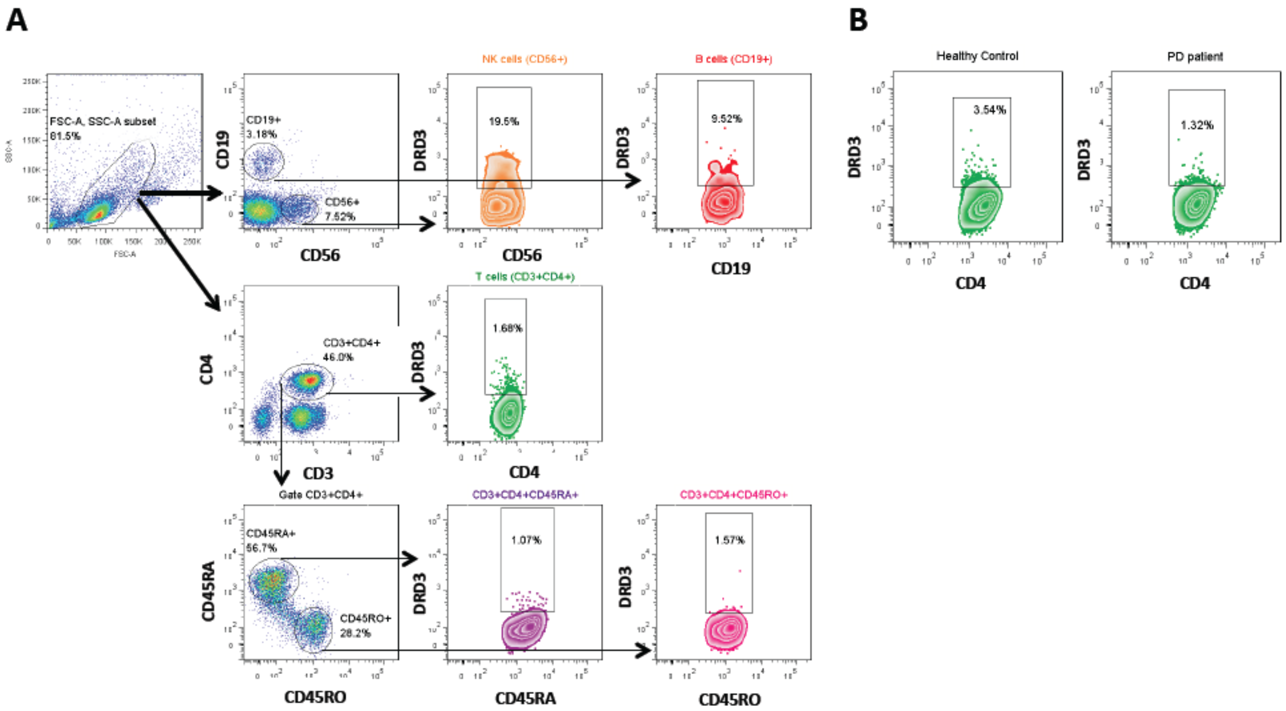

Supplementary Figure 1. Gating strategy for flow cytometry analysis in human cells. PBMCs were isolated from healthy controls (HC) or PD patients and then immunostained for several surface markers and for dopamine receptor D3 (DRD3). Different lymphocyte populations and their DRD3 expression were analysed by flow cytometry. (**A**) Cells with characteristic lymphoid FSC-A and SSC-A distribution were gated and stained for CD19 and CD56 (upper panels) or CD3 and CD4 (middle panels). DRD3 expression was analysed in NK cells (gate $CD56^{+}CD19^{-}$), in B cells (gate $CD19^{+}CD56^{-}$) and in CD4+ T-cells (gate $CD3^{+}CD4^{+}$). In addition, total $CD4^{+}$ T-cells were analysed for subsets of naïve cells ($CD45RA^{+}CD45RO^{-}$) and memory cells ($CD45RA^{-}CD45RO^{+}$) as shown in bottom panels. Representative dot-plots and density plots are shown. (**B**) Representative density plots of DRD3 expression in the $CD3^{+}CD4^{+}$ T-cell population obtained from healthy controls and PD patients.

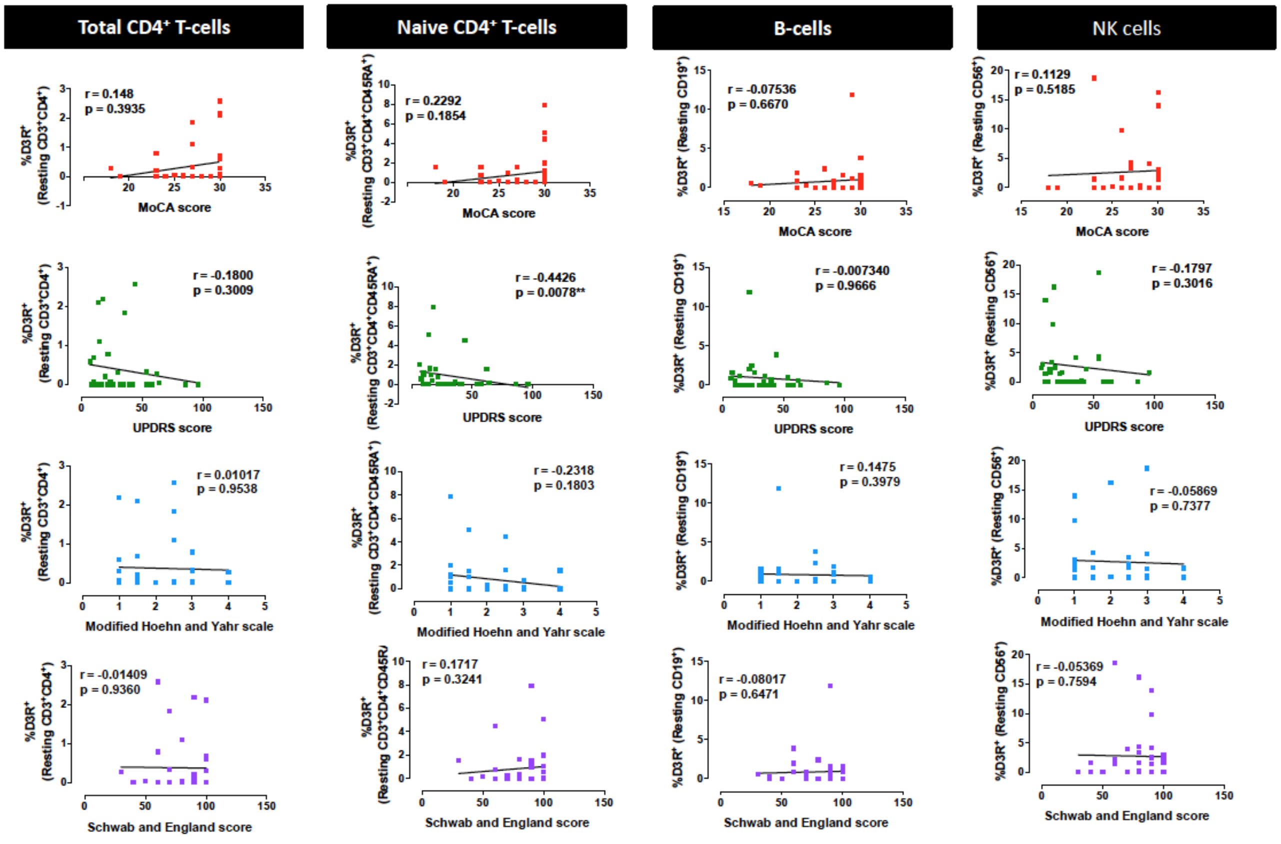

Supplementary Figure 2. Correlation analysis of DRD3 expression in lymphocytes and disease activity in PD patients. PBMCs were isolated from PD patients (n = 41) and then immediately immunostained for several surface markers and for dopamine receptor D3 (DRD3). Different lymphocyte populations and their DRD3 expression were analysed by flow cytometry. DRD3 expression was quantified as the frequency of DRD3$^+$ cells in total CD4$^+$ T-cells (gate CD3$^+$CD4$^+$), naive CD4$^+$ T-cells (gate CD3$^+$CD4$^+$CD45RA$^+$CD45RO$^-$), B-cells (gate CD19$^+$CD56$^-$) and NK cells (gate CD56$^+$CD19$^-$). Disease activity was quantified as the MoCA score (red symbols), UPDRS score (green symbols), modified Hoehn and Yahr scale (blue symbols) or Schwab and England score (purple symbols) and represented versus DRD3$^+$ frequency in different lymphocyte populations. Correlation was calculated by the Spearman test. **, p<0.01.

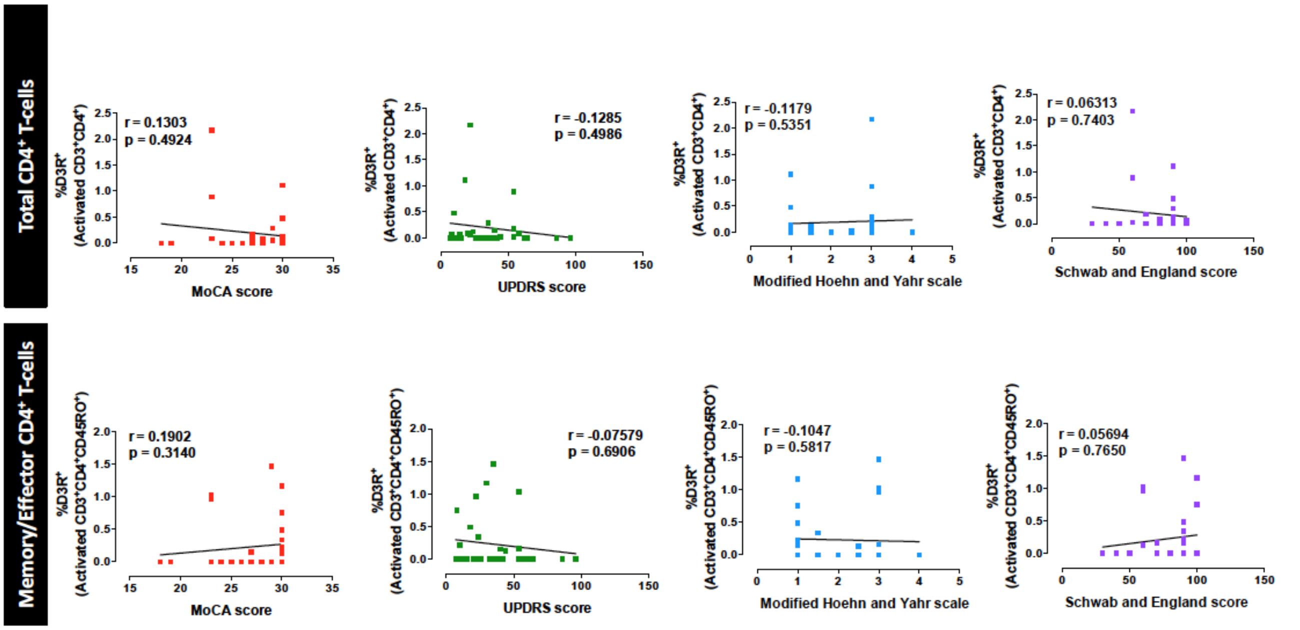

Supplementary Figure 3. Correlation analysis of DRD3 expression in activated CD4$^+$ T-cells and disease activity in PD patients. PBMCs were isolated from PD patients (n = 41), activated with anti-CD3 and anti-CD28 antibodies for 72 h and then immunostained for several surface markers and for dopamine receptor D3 (DRD3). DRD3 expression was analysed by flow cytometry. DRD3 expression was quantified as the frequency of DRD3$^+$ cells in and in total CD4$^+$ T-cells (gate CD3$^+$CD4$^+$) and memory/effector CD4$^+$ T-cells (gate CD3$^+$CD4$^+$CD45RA$^-$CD45RO$^+$). Disease activity was quantified as the MoCA score (red symbols), UPDRS score (green symbols), modified Hoehn and Yahr scale (blue symbols) or Schwab and England score (purple symbols) and represented versus DRD3$^+$ frequency in different lymphocyte populations. Correlation was calculated by the Spearman test. No significant differences were found.

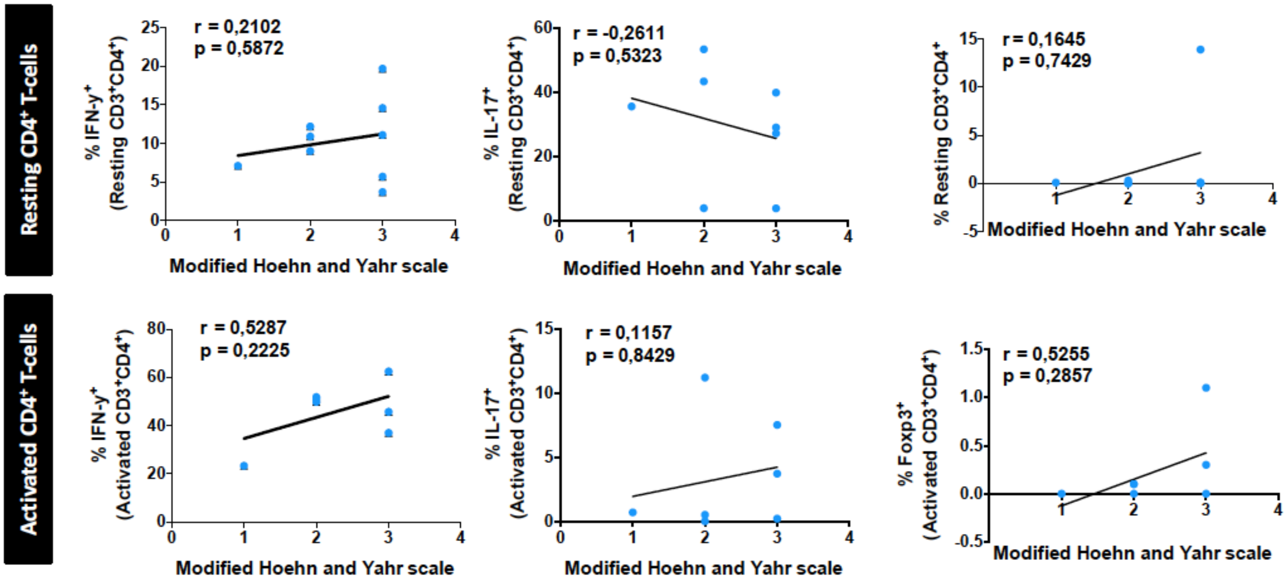

Supplementary Figure 4. Correlation analysis of phenotypic profile of activated CD4^{+} T-cells and disease activity in PD patients. PBMCs were isolated from PD patients (n = 9), and immediately analysed (top panels) or activated with anti-CD3 and anti-CD28 antibodies for 72 h (bottom panels). To analyse the profile of T-cell phenotype, cells were stimulated with PMA and ionomycin in the presence of brefeldin A for 4h and IFN-γ (left panels), IL-17 (middle panels) and Foxp3 (right bottom panel) expression was determined by intracellular immunostaining in the CD3^{+} CD4^{+} gated population. The frequency of CD3^{+} CD4^{+} cells from total PBMCs was also determined in resting conditions (right top panel). Cells were then analysed by flow cytometry. The percentage of Th1, Th17 and Treg cells was respectively quantified as the frequency of IFN-γ^{+} , IL-17^{+} and Foxp3^{+} cells in the gate CD3^{+}CD4^{+}. Disease activity was quantified as the modified Hoehn and Yahr scale and represented versus the percentage of IFN-γ^{+} , IL-17^{+}, Foxp3^{+} or total CD3^{+} CD4^{+} cells. Correlation was calculated by the Pearson (when data was normally distributed) or Spearman (when data was not normally distributed) tests. No significant differences were found.

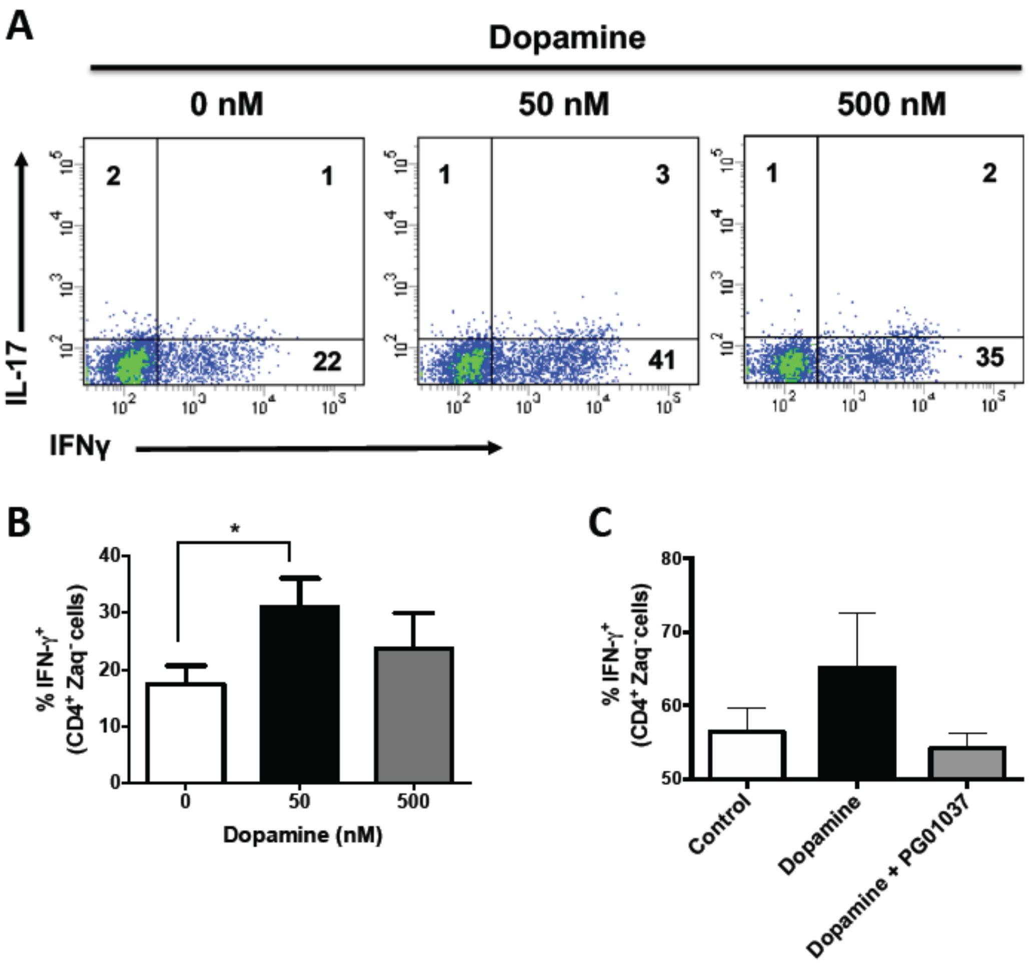

Supplementary Figure 5. Selective DRD3 stimulation on CD4$^+$ T-cells favours Th1-differentiation. Naive CD4$^+$CD25$^-$ T-cells obtained from WT mice were cultured under Th1 conditions for 4 days and cytokine production was evaluated by flow cytometry. (A and B) Naive T-cells were co-treated with 0, 50 nM or 500 nM dopamine and cultured in the presence of plate-bound anti-CD3 and a cocktail of soluble antibodies and cytokines (see Materials and Methods) for 4 days. Then, cells were restimulated with PMA/Ionomycin in the presence of Brefeldin A for 4 hours and intracellular cytokine analysis was carried out by flow cytometry. (**A**) representative dot plots for IL-17 versus IFNγ production in the alive (Zaq$^-$) CD4$^+$ population are shown. Numbers indicate the percentage of IFNγ$^+$ (bottom right), IFN-γ$^+$ IL-17$^+$ (right top) and IL-17$^+$ (top left) cells, respectively. (**B**) Quantification of the percentage of CD4$^+$ T-cells producing IFN-γ. Values represent mean ± SEM from three independent experiments. *, $p<0.05$. (**C**) Naive T-cells were left untreated or pre-incubated with 20 nM PG01037 and then treated with 50 nM dopamine and cultured in the presence of plate-bound anti-CD3 and a cocktail of soluble antibodies and cytokines (see Materials and Methods) for 4 days. Cells were restimulated with PMA/Ionomycin in the presence of Brefeldin A for 4 hours and intracellular cytokine analysis was carried out by flow cytometry. The quantification of the frequency of IFNγ+ cells in the alive (Zaq$^-$) CD4$^+$ population are shown. Values represent mean ± SEM from two independent experiments.

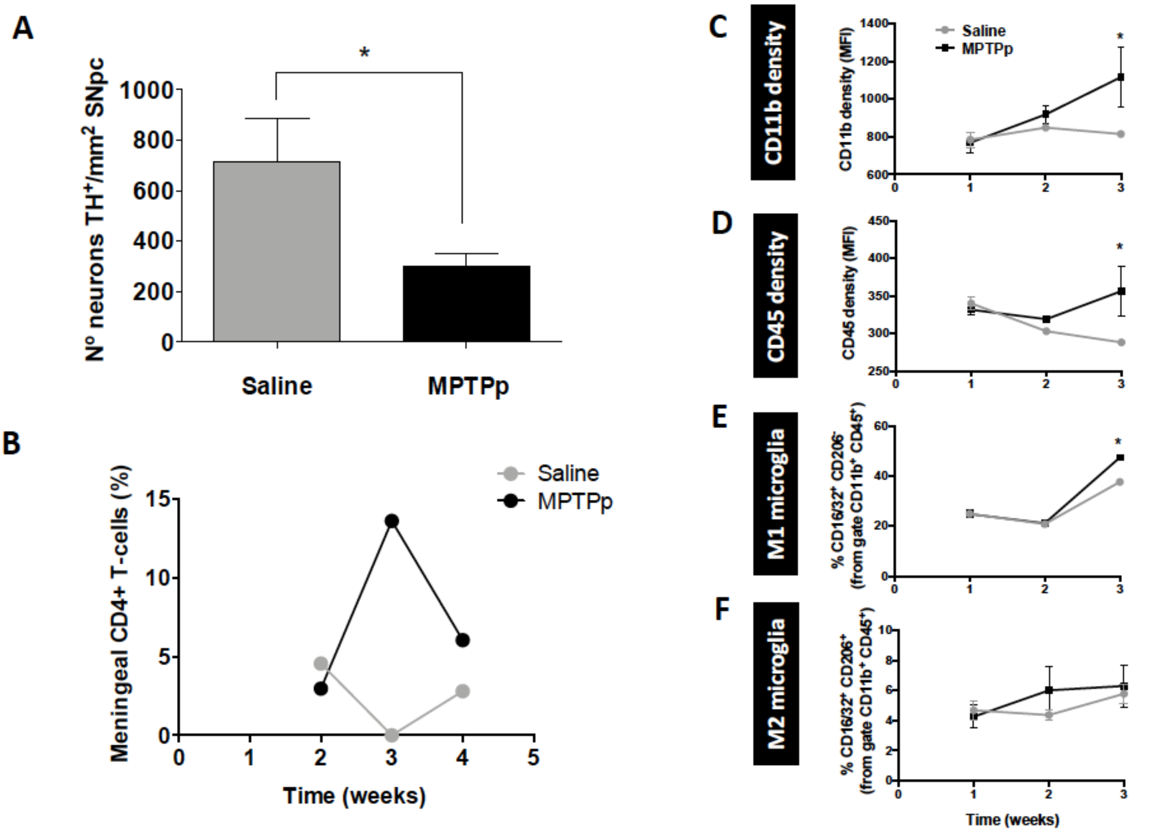

Supplementary Figure 6. Kinetic analysis of T-cell infiltration and neuroinflammation in MPTPp-treated mice. Control animals (without MPTP treatment) were treated with saline and probenecid. MPTPp animals were injected with MPTP (20 mg/kg) and probenecid (250 mg/kg). All compounds were administered by intraperitoneal injections following the experimental scheme indicated in figure 2A. Animals were sacrificed at different time points, transcardially perfused with PBS and then brains and meningeal lymphatics were obtained. Samples were digested with collagenase and DNAse and immunostained for subsequent flow cytometry analyses. (**A**) some experimental mice in the control and in the MPTPp groups were sacrificed after 5 weeks of MPTPp or saline administration and the extent of TH^+ neurons in the SNpc was quantified by immunohistochemistry. (**B**) Frequency of $CD4^+$ T-cells infiltrating meningeal lymphatics was analysed by flow cytometry. Cells from 3-5 mice per group were pooled and analysed. (C-F) $CD11b^+$ $CD45^+$ cells were selected from the gate of living cells (Zaq^- population) in brain samples and then CD16/32 and CD206 expression was analysed to determine M1 and M2 phenotypes. Density of CD11b (**C**) and CD45 (**D**) surface expression was determined throughout the time-course of MPTPp-intoxication. Frequencies of M1 (**E**) and M2 (**F**) phenotypes were also determined throughout the time-course of MPTPp-intoxication. M1 and M2 microglial phenotypes were defined as $CD16/32^+$ $CD206^-$ cells and $CD16/32^+$ $CD206^+$ cells respectively in the $CD45^+$ $CD11b^+$ population. Values represent mean ± SEM. Data from 3 to 5 mice per group is shown. *, $p<0.05$ by Student's t-test.

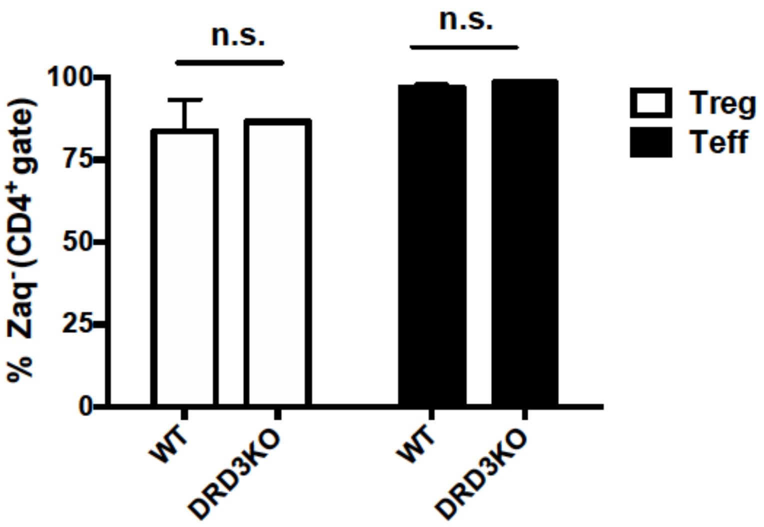

Supplementary Figure 7. DRD3 deficiency does not affect CD4+ T-cell viability. GFP^+ (Treg) and GFP^- (Teff) $CD3^+$ $CD4^+$ T-cells were isolated from WT or DRD3KO *Foxp3*^{gfp} reporter mice and activated with anti-CD3 and anti-CD28 antibodies for 6 days. Viability was analysed using zombieaqua (Zaq) viability kit. The frequency of Zaq- cells in the CD4+ T-cell population was quantified. A representative of several experiments performed in triplicated is shown. Values are mean ± SEM. n.s., non-significant differences were found.

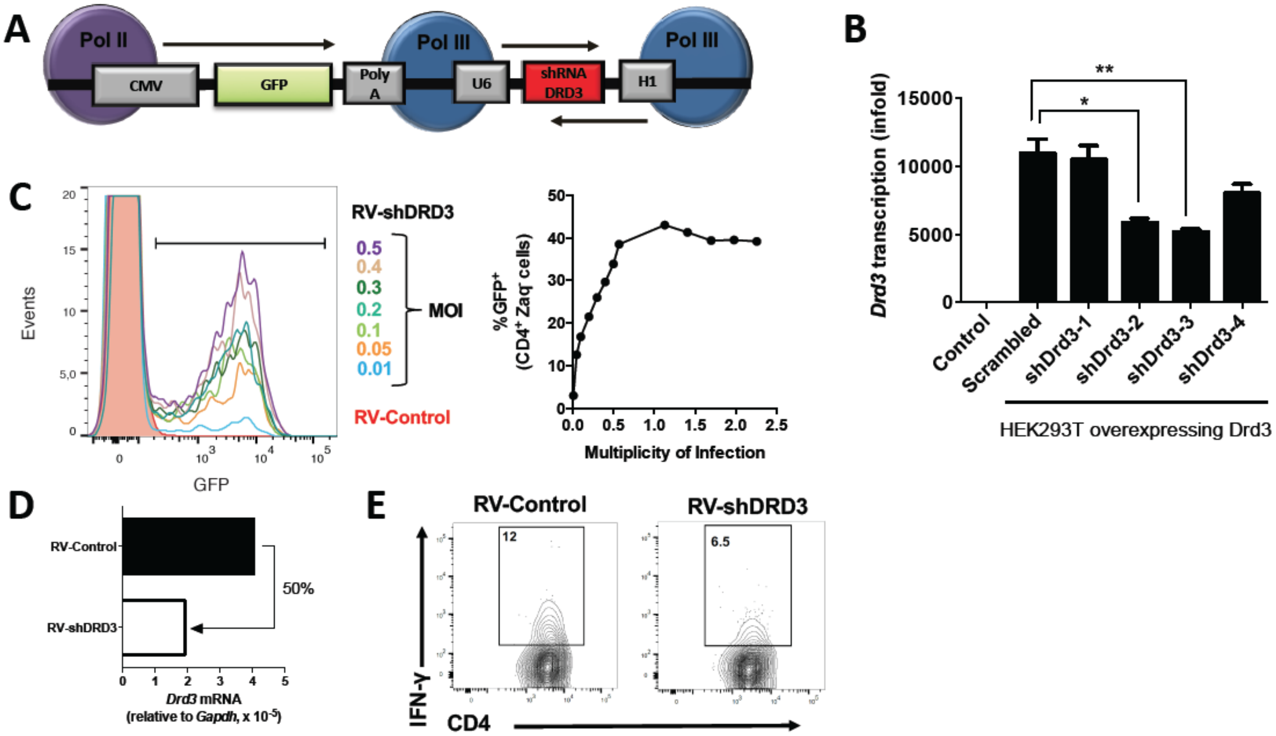

Supplementary Figure 8. Silencing of DRD3 expression on CD4$^+$ T cells impairs Th1 phenotype. (**A**) Scheme of the construct codifying for shRNA for DRD3 (shDRD3). The construct codify for the expression of shRNA under the control of promoters U6 and H1 and to the expression of the reporter gene green fluorescent protein (GFP) under the control of the promoter for cytomegalovirus (CMV). (B and D) Wild type naïve CD4$^+$ T-cells were activated *in vitro* under Th1-polarizing conditions for 48 hours and then transduced with retrovirus codifying for shDRD3 for 48 hours. Cells transduced with control retrovirus codifying just for a reporter gene (RV-Control) were included as controls. (**B**) HEK293T cells were non-transduced (control) or transduced with lentiviral particles codifying for Drd3 and RFP under the control of CMV promoter, and RFP+ cells were purified by cell-sorting (HEK293T cells overexpressing Drd3). Subsequently, cells were transduced with different shRNA for Drd3 (shDrd3 1-4) for 24h and the levels of Drd3 transcripts were determined by qRT-PCR. Levels of *Gfap* transcripts were used as house-keeping. Data was normalised with control HEK293T cells (infold). Values are mean ± SEM. *, p<0.05; **, p<0.01 by one-way ANOVA followed by Tukey's *posthoc* test. shDrd3-3 was selected for subsequent experiments. (**C**) Cells transduced with RV-shDRD3 at different multiplicities of infection (MOI) were analysed for GFP expression on the alive (Zaq$^-$) CD4$^+$ population. Representative histograms for GFP expression are shown in the left panel. The frequency of GFP$^+$ cells in the alive CD4$^+$ T-cell population is shown in the right panel. (**D**) *Drd3* mRNA transcription was quantified by qPCR on sorted RV-Control and RV-shDRD3 transduced cells. (**E**) Representative contour plots showing IFN-γ production by transduced CD4$^+$ T-cells are shown. Numbers in quadrants indicate the frequency of IFN-γ$^+$ among transduced CD4$^+$ T-cells.

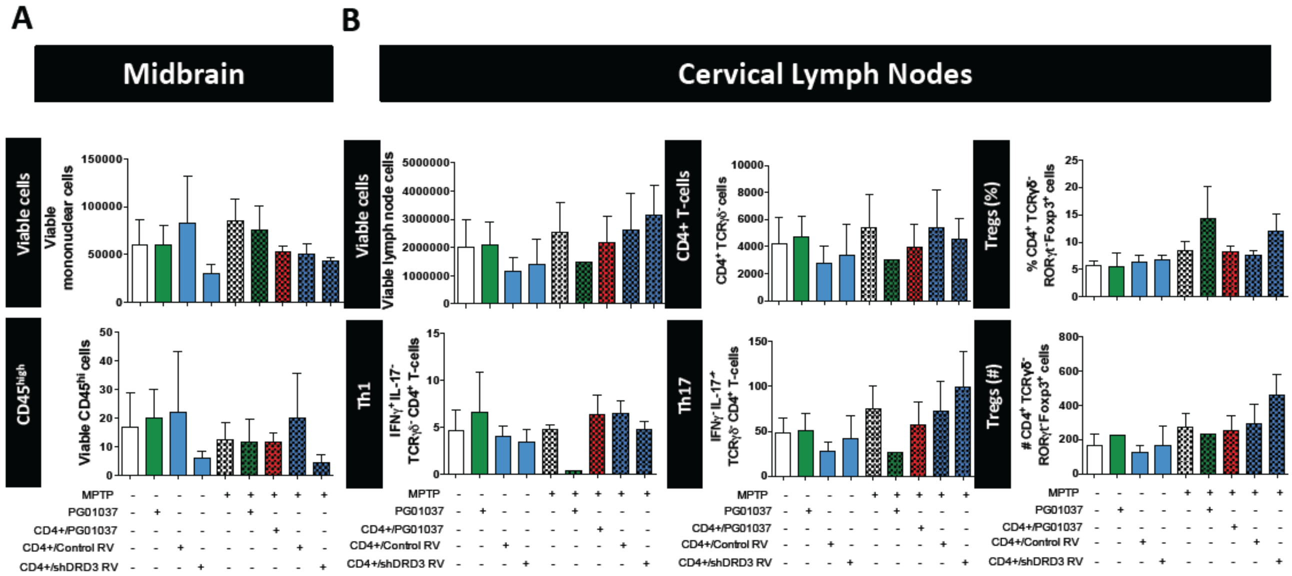

Supplementary Figure 9. Comparison of T-cell infiltration into the brain and cervical lymph nodes in MPTPp-intoxicated mice upon treatment with systemic DRD3-antagonistm or the intravenous transference of $CD4^+$ T-cells transduced with shRNA for DRD3 or treated with a DRD3 antagonist. Animals were treated as described in figure 5A and sacrificed after three weeks of MPTPp intoxication (at the end of week number 4 in the scheme of figure 5A). The infiltration of different inflammatory and anti-inflammatory lymphocyte subsets into the midbrain (**A**) and cervical lymph nodes (**B**) were analysed by flow cytometry. Absolute numbers (A and left, middle and bottom right panels from B) and frequency (upper right panel from B) per animal of different lymphocyte subsets obtained from the different experimental groups were quantified. Data represent the mean with the SEM. One-way ANOVA followed by Tukey's multiple comparison post-hoc test were used to determine statistical differences: No significant differences were detected; n = 2-4 mice per group.

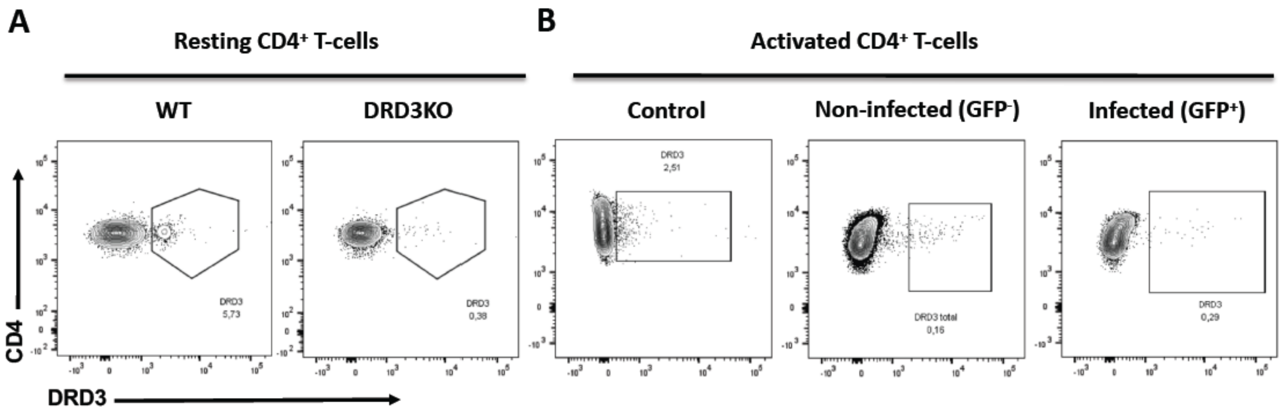

Supplementary Figure 10. DRD3 expression in resting and activated CD4$^+$ T-cells and upon shDRD3-transduction. (**A**) Total CD4$^+$ T-cells were isolated from the spleen of WT or DRD3KO mice and immediately immunostained with anti-CD4 antibody and anti-DRD3 antibody (ADR-003, Alomone Labs) and analysed by flow cytometry. (**B**) Total CD4$^+$ T-cells were isolated from the spleen of WT mice and activated with anti-CD3 and anti-CD28 antibodies for 48h in the absence (Control) or in the presence of RV-shDRD3. Afterward, cells were immunostained with anti-CD4 antibody and anti-DRD3 antibody (ADR-003, Alomone Labs) and analysed by flow cytometry. When cells were treated with RV-shDRD3, infected (GFP$^+$) and non-infected cells (GFP$^-$) were analysed separately. (A and B) Representative contour-plots for CD4 and DRD3 immunostaining in the Zaq$^-$ gate are shown. Numbers on contour-plots indicate the percentage of DRD3$^+$ cells.

Table S1. Summary of the therapeutic effect observed for different treatments in mice intoxicated with MPTPp*

	TEST	COAT-HANGER TEST		BEAM TEST	NEURODEGENERATION		NEUROINFLAMMATION
	PARAMETER***	# Sections	Extreme latency (sec)	# Errors	TH+ in SNpc	DAT+ in Striatum	Iba1(high) in striatum
Experiment**							
2	i.p. PG01037 (30 mg/kg)	Yes (p<0.0001)	Invalid	Yes (p<0,05)	Yes (p<0,05)	n.s.	Yes (p<0.0001)
1	i.v. CD4+ T-cells (4x105 cells)	Invalid	n.s.	n.s.	n.s.	Yes (p<0,05)	n.d.
1	i.v. CD4+ T-cells (4x105 cells) + PG01073 (20 nM)	Invalid	Yes (p<0,001)	Yes (p<0,05)	n.s.	n.s.	n.d.
2	i.v. CD4+ T-cells (4x105 cells) + PG01073 (20 nM)	Yes (p<0.01)	Invalid	Yes (p<0,05)	n.s.	n.s.	Yes (p<0.001)
1	i.v. CD4+ T-cells (7x105 cells) + PG01073 (20 nM)	Invalid	n.s.	n.s.	n.s.	n.s.	n.d.
1	i.v. CD4+ T-cells (10x105 cells) + PG01073 (20 nM)	Invalid	n.s.	n.s.	n.s.	n.s.	n.d.
1	i.v. CD4+ T-cells (4x105 cells x 3 injections) + PG01073 (20 nM)	Invalid	n.s.	n.s.	n.s.	n.s.	n.d.
2	i.v. CD4+ T-cells (4x105 cells) + RV-Control (MOI 1)	n.s.	Invalid	n.s.	n.s.	n.s.	n.d.
2	i.v. CD4+ T-cells (4x105 cells) + RV-shDRD3 (MOI 1)	n.s.	Invalid	n.s.	n.s.	n.s.	n.d.

* Improvement versus MPTPp group; ** Experiment 1 is shown in figures 2-4 and experiment 2 is shown in figures 5-7;***Parameter is considered invalid when the MPTPp group is not significantly different to the heathy control groups in corresponding experiment. Not significant: n.s.; Not determined: n.d.
